# Role of Ionizing Radiation in Shaping the Complex Multi-Layered Epigenome

**DOI:** 10.3390/epigenomes9030029

**Published:** 2025-08-08

**Authors:** Claudia E. Rübe, Mutaz A. Abd Al-razaq, Carola Meier, Markus Hecht, Christian Rübe

**Affiliations:** 1 Department of Radiation Oncology, Saarland University Medical Center, 66421 Homburg, Germany; mutaz.abd-al-razaq@uks.eu (M.A.A.A.-r.); markus.hecht@uks.eu (M.H.);; 2 Department of Anatomy and Cell Biology, Medical Faculty, Saarland University, 66421 Homburg, Germany; carola.meier@uni-saarland.de

**Keywords:** ionizing radiation, radiation-induced DNA damage, epigenetic dysfunction, premature senescence, radiation reactions

## Abstract

The impact of ionizing radiation (IR) with induction of various DNA damage is based not only on genetic but also on epigenetic effects. Epigenetic modifications determine the chromatin structure and DNA accessibility, thereby regulating cellular functions through the expression of individual genes or entire groups of genes. However, the influence of DNA repair processes on the restoration of local chromatin structures and global nuclear architectures is still insufficiently understood. In multicellular organisms, epigenetic mechanisms control diverse cellular functions of specific cell types through precise temporal and spatial regulation of gene expression and silencing. How altered epigenetic mechanisms regulate the pathophysiological function of cells, tissues, and ultimately entire organs following IR exposure remains to be investigated in detail. Radiation-induced epigenetic processes are particularly critical for immature cell populations such as tissue-specific stem and progenitor cells during development and differentiation of organ tissues. Genome-wide patterns of DNA and histone modifications are established cell types—specifically during the development and differentiation of organ tissues but can also be fundamentally altered in adult organism by stress responses, such as radiation-induced DNA damage. Following IR exposure, epigenetic factors are not always fully restored to their original state, resulting in epigenetic dysfunction that causes cells to lose their original identity and function. Moreover, severe radiation-induced DNA damage can induce premature senescence of cells in complex tissues, which ultimately leads to signs of aging and age-related diseases such as cancer. In this work, we provide an overview of the most important epigenetic changes following IR exposure and their pathophysiological significance for the development of acute and chronic radiation reactions.

## 1. Chromatin Organization

In the nuclei of all eukaryotic cells, genomic DNA is highly folded, constrained, and compacted by histone and non-histone proteins in a dynamic polymer called chromatin. The distinct levels of chromatin organization are dependent on the dynamic higher-order structuring of nucleosomes, which represent the basic repeating unit of chromatin [1]. In each nucleosome, roughly two superhelical turns of DNA around an octamer of core histone proteins formed by four histone partners: an H3-H4 tetramer and two H2A-H2B dimers. Histones are small basic proteins consisting of globular domains and more flexible and charged NH2 termini (histone “tail”) that protrude from the nucleosome. Moreover, there are structurally different histone variants with special epigenetic functions [2]. Nucleosomes are connected by linker DNA and stabilized by histone protein H1, modulating the distance between neighboring nucleosomes. The eukaryotic genome is packaged in nucleosomal beads-on-a-string architecture, thereby protecting, condensing, and organizing the long but thin DNA fiber in the nucleus [1]. However, DNA acts as template for numerous nuclear processes that require access to genetic information and therefore open chromatin conformations. A number of mechanisms have evolved to counteract the primarily repressive nature of the nucleosome, permitting flexible and responsive nuclear activities. In interphase nucleus, chromatin exists as either loose, transcriptionally active euchromatin or dense, transcriptionally inactive heterochromatin. Chemical alterations to histone proteins can induce the formation of either the open euchromatin state, which facilitates gene expression by allowing transcription factors and enzymes to interact with DNA, or the closed heterochromatin state, which suppresses gene expression by preventing initiation of transcription. The organizational structure of chromatin thus modulates the access of transcription factors and RNA polymerase to DNA promoters, and therefore contributes the fluctuating status of gene expression patterns [3]. Overall, epigenetics provides a fundamental regulatory system for DNA packaging rules whose functional significance goes far beyond the sequence of information of our genetic code. Especially after stress reactions such as radiation-induced DNA damage, regulatory chromatin modifications determine complex cell functions through their fine-tuned gene regulation and ultimately have decisive influence on radiation sensitivity.

## 2. Epigenetic Processes

### 2.1. Epigenetic Writers and Erasers in Chromatin Dynamics

Epigenetic regulation is a dynamic process, and reversible modifications of DNA and histones have emerged as key players in the regulation of gene expression. Epigenetic writers are enzymes that add chemical groups to DNA or histone proteins, thereby modifying chromatin structure and influencing the recruitment of DNA repair proteins. Histone acetyltransferases (HATs) acetylate histones (e.g., H4K16ac), promoting chromatin relaxation, while histone methyltransferases (HMTs) generate repressive marks (H3K9me3; H3K27me3) and DNA methyltransferases (DNMTs) can induce long-term silencing of DNA regions. Ubiquitination and sumoylation further modulate chromatin structure and protein activity, with ubiquitin often marking proteins for degradation or altering their function and sumoylation generally repressing gene expression. PARP enzymes play a crucial role in DNA repair by adding ADP-ribose polymers to proteins, relaxing chromatin around damage sites. Epigenetic erasers, such as histone deacetylases and demethylases, as well as sumoylation and ubiquitination proteases regulate the removal of these modifications, ensuring proper control of chromatin states. Together, these different classes of enzymes form a highly dynamic and coordinated system of “writers” and “erasers” for controlling chromatin structure and gene expression to modulate cellular processes in response to genotoxic stress.

### 2.2. DNA Methylation of CpG-Rich DNA Sequences

Catalyzed by DNA methyltransferase enzymes, DNA methylation involves the addition of methyl groups to cytosine nucleotides within cytosine–guanine (CpG) sequences, which are often surrounded by other CpGs, forming CpG islands. Most of the gene promoter regions are located within CpG islands and are frequent targets of epigenetic DNA methylation. Methylated cytosines in promoter regions recruit gene suppressor proteins and reduce the interaction between DNA and transcription factors, leading to gene silencing. Cytosine methylation also promotes the formation of heterochromatin, so that tightening of the nucleosome prevents the transcription machinery from interacting with DNA. During development, the pattern of DNA methylation in the genome changes through a dynamic process involving both de novo DNA methylation and demethylation. As a result, differentiated cells develop a stable and unique DNA methylation pattern that regulates tissue-specific gene transcription [4].

### 2.3. Post-Translational Modifications

Enzyme-catalyzed acetylation, methylation, phosphorylation, or ubiquitination are among the post-translational modifications (PTMs) of histone proteins, each of which alters DNA–histone interactions in nucleosomes [5]. Histone acetylation often occurs at positively charged lysine residues, which weakens DNA–histone interactions, thus opening the chromatin and facilitating transcription. For example, acetylation of lysine 9 and lysine 27 on histone 3 (H3K9ac and H3K27ac, respectively) correlates with transcription activation. Histone methylation is more complex as it does not change the histone protein charge and can include the addition of 1–3 methyl groups to lysine and 1–2 methyl groups to arginine. For example, methylation of lysine 4 on histone 3 (H3K4me) is associated with transcription activation, while trimethylation of lysine 27 on histone 3 (H3K27me3) correlates with transcription repression. Histone phosphorylation involves the addition of negative phosphate groups to histone tails, but less is known of its function aside from phosphorylation of H2AX playing a role in the DNA damage response [6]. The molecular choreography of the DNA damage response (DDR) relies also on PTMs such as ubiquitylation, SUMOylation, and poly(ADP-ribosyl)ation to regulate DNA damage signaling and repair. These damage-induced, chain-like PTMs contribute to the multi-protein assemblies found at sites of DNA damage and regulate their spatio-temporal dynamics. Aside from the relatively straightforward effect of histone acetylation on gene expression, the effects of other PTMs are complex and greatly influenced by the state of nearby DNA molecules. According to the histone code hypothesis, PTMs act as epigenetic marks for certain biological processes such as transcription, replication, recombination, or repair [7].

### 2.4. Non-Coding RNA-Associated Gene Silencing

Non-coding RNA are functional RNA molecules that are transcribed but not translated into proteins. Once regarded as waste of the genome, recent insight suggests that non-coding RNA molecules are key players in cell regulatory networks of epigenetic gene expression [8]. Notable non-coding RNA molecules include microRNAs (miRNA), short interfering RNAs (siRNA), which both include less than 30 nucleotides, and long non-coding RNAs (lncRNA), which are 200 nucleotides or longer. As fundamental evolutionary strategy, non-coding RNA molecules enable the timely and reversible control of mammalian gene expression. RNA-mediated epigenetic regulation profoundly influences development and tissue homeostasis, and dysfunctional alterations can contribute to the development and progression of various human diseases, particularly cancer. An overview of the diversity of targets, interaction partners, and mechanisms involved in RNA-mediated modulation of epigenetic states is provided in the following review [8].

### 2.5. Replacement of Core Histones with Histone Variants

The incorporation of specialized histone variants can modulate the stability of nucleosomal structures, and the degree of nucleosomal packaging can lead to profound changes in the chromosomal superstructures in which DNA is packaged. Nucleosome positioning and chromatin compaction can have profound consequences on all DNA-mediated processes, including gene regulation. Modified chromatin structures following IR exposure can alter DNA accessibility and thereby regulate the pattern of gene expression. In contrast to canonical histone proteins, histone variants are produced by different histone genes throughout the cell cycle. This gives epigenetic processing an additional degree of flexibility and complexity and can therefore influence a wide range of biological processes even in post-mitotic cells. Among the core histones, the H2A family shows the highest sequence divergence, resulting in the largest number of known variants [9]. Even though histone variants may differ by only few amino acids relative to their canonical counterparts, these minor variations can profoundly alter chromatin structure, accessibility, dynamics, and gene expression. Histone variants often interact with dedicated chaperones and remodelers and can have unique PTMs that shape gene expression landscapes. Histone variants also play essential roles in DNA replication, damage repair, and histone–protamine transition during spermatogenesis [9].

## 3. Epigenetic Processes Following Exposure to Ionizing Radiation

### 3.1. Ionizing Radiation and DNA Damage Induction

The human body can tolerate certain levels of IR without immediate harm, but exceeding recommended limits increases the risk of both acute and long-term health effects. IR has very heterogeneous effects on different organs because each tissue varies in its cellular composition and thus radiosensitivity. Rapidly dividing tissues like blood-forming bone marrow, gastrointestinal lining, and skin are more vulnerable and can suffer acute effects due to cell damage or cell death caused mainly by breaking of chemical bonds in DNA. In contrast, tissues with slower cell turnover such as muscle or nerve tissue are generally less acutely affected but can still experience long-term effects like radiation-induced fibrosis or increased cancer risk. Overall, the impact of radiation depends on the tissue’s characteristics, making its effects quite diverse across the body. To account for biological effects, the radiation dose is usually measured in Sievert (Sv), whereas in radiotherapy, the absorbed dose is expressed in Gray (Gy). Strict safety guidelines protect individuals from harmful exposure in public (≤1 mSv/year) and occupational (≤20 mSv/year) settings, but high doses of IR, such as those used in radiotherapy, can have significant and often harmful effects not only on tumor tissue but also on surrounding healthy tissue. The risk of damaging normal tissues often limits the maximum radiation dose that can be safely delivered. The goal of radiotherapy is to maximize the therapeutic ratio, which is the balance between tumor control and normal tissue complications. The specific dosage for a full course of radiotherapy varies considerably depending on the type and stage of cancer being treated and the specific treatment plan but generally requires total doses between 50 and 80 Gy for successful tumor treatment. Researchers are constantly working to improve radiation delivery techniques and develop strategies to protect normal tissues, thereby improving the effectiveness and safety of cancer treatment.

IR causes different types of DNA damage, with the repair of DNA double-strand breaks (DSBs) being crucial for cell survival. In recent decades, extensive studies have investigated how radiation-induced DNA damage is repaired through complex DDR mechanisms to restore genomic integrity [10]. DSBs are repaired by different pathways, mainly by non-homologous end-joining (NHEJ) and homologous recombination (HR). The balance of these signaling pathways depends on the local chromatin context, but the underlying mechanisms of the required chromatin modeling are poorly understood [11]. Moreover, the impact of these DNA repair processes on epigenetic control mechanisms and physiological functions of cells, tissues, and organs, and ultimately on radiation-related health effects, are still largely unknown. In multicellular organisms, epigenetic mechanisms control cellular functions through complex and precise temporal and spatial regulation of gene expression and silencing. Radiation-induced epigenetic processes are particularly critical for immature cell populations such as tissue-specific stem and progenitor cells during organ tissue development and differentiation [12]. Epigenetic modifications lead to variations in chromatin condensation, which regulates the expression of individual genes or entire gene groups and thus determines the unique functions of specific cell types. Genome-wide patterns of DNA and histone modifications are established cell type-specifically during the development and differentiation of organ tissues but they can also be fundamentally altered in the adult organism by stress responses, such as DNA damage following IR exposure. The repair of radiation-induced DNA damage occurs in the complex chromatin of the cell nucleus and requires structural changes in chromatin architecture in specific temporal and spatial sequences. After successful repair of radiation-induced DNA damage, the chromatin organization must be fully reestablished to restore the original cell function [3]. This requires not only the assembly of histones into nucleosomal structures, but also the precise restoration of the higher-order structure of the chromatin fiber in the nucleus defined by multiple epigenetic processes [1]. Since higher-level epigenetic control elements regulate cellular proliferation and differentiation, they play also crucial roles in the regeneration of organ tissues following IR exposure, and thereby decisively influence radiation reactions [12]. The main epigenetic changes currently under investigation are DNA methylation, histone modification, and modulation of non-coding RNAs. The incorporation of histone variants has recently been described as another important epigenetic mechanism in the radiation response. Collectively, these epigenetic mechanisms appear to bridge the gap between genotype and phenotype by regulating gene function.

### 3.2. Chromatin Remodelers During Acute DNA Damage Response

One of the earliest and most critical steps in acute DDR is the remodeling of chromatin to allow access for DNA repair machinery. This remodeling is orchestrated by ATP-dependent chromatin remodelers in close coordination with histone-modifying enzymes which alter the interaction within or between neighboring nucleosomes and recruit chromatin-binding proteins to specific regions [13]. Among the key chromatin remodelers involved are the SWI/SNF complexes capable of altering chromatin organization through sliding nucleosomes or evicting histones from chromatin. The SWI/SNF family plays a central role in chromatin remodeling at sites of DNA damage and contributes to successful repair in both HR and NHEJ by promoting access of essential repair proteins to damaged DNA [14]. A well-charaterized change in chromatin organization is the rapid formation of open chromatin structures by increased acetylation of histone tails on nucleosomes at DSBs [15]. Acetylation of histone H2AX and H4 is dependent on the Tip60 acetyltransferase, weakening nucleosome interactions and promoting dynamic binding of repair factors to damaged chromatin [16]. The INO80 complex is another critical chromatin remodeler activated in response to IR. INO80 is recruited to sites of damage through interactions with γH2AX and other DNA damage mediators [17]. Unlike SWI/SNF, INO80 specializes in nucleosome sliding and histone variant exchange, processes that are particularly important for HR repair [18]. INO80 promotes DNA end resection, a necessary step in HR, and also aids in restoring chromatin structure following repair. It coordinates with histone deacetylases and methyltransferases to dynamically regulate histone modifications during and after the repair process. Together, chromatin remodelers and histone modifiers act in a highly coordinated manner to modulate chromatin architecture in response to DNA damage. While remodelers like SWI/SNF and INO80 physically reposition nucleosomes, histone modifiers alter histone marks—such as acetylation, methylation, and phosphorylation—to signal damage, recruit repair factors, and ultimately reestablish normal chromatin states. This integrated response ensures both effective DNA repair and the preservation of chromatin integrity post-damage [19].

### 3.3. Ubiquitylation, SUMOylation, and PARylation During DNA Damage Response

DSB formation triggers rapid, hierarchical recruitment of numerous signaling and repair factors into nuclear foci lining an extensive chromatin region around individual lesions. This local enrichment of many genome maintenance factors near DSBs is driven by PTMs of the adjacent chromatin and attracted factors to ensure efficient DSB repair. Together, these multiple PTMs have crucial and widespread roles in promoting the complex cellular responses in diverse chromatin context to re-establish genome integrity after DSBs [20] (Table 1).

#### 3.3.1. Ubiquitylation

Exposure to IR triggers a cascade of ubiquitylation events that modify chromatin and facilitate DNA repair. For the repair of DSBs, the choice between NHEJ and HR is tightly controlled, and imbalances in their regulation can contribute to genome instability [45]. Recruitment of key mediators triggering DSB repair pathway choice critically depends on local ubiquitin conjugations at DNA break sites [23,27,46]. Generally, the formation of covalently linked ubiquitin–protein conjugates occurs via multi-step enzymatic cascades involving ubiquitin-activating (E1) and ubiquitin-conjugating enzymes (E2) as well as ubiquitin ligases (E3), which together transfer the ubiquitin chain to lysine residues in target proteins. These modifications are recognized by proteins with ubiquitin-binding domains and can be removed by ubiquitin-specific proteases [27]. Chromatin ubiquitination by RNF8, RNF168, and other E3 ubiquitin ligases can lead to complex ubiquitination landscapes at DSB sites, thereby promoting the accumulation of multiple DNA repair factors near the lesions. Upon DSB induction, the E3 ubiquitin ligases RNF8 and RNF168 are recruited to chromatin and catalyze the formation of K63-linked ubiquitin chains on histones H2A and H2AX, creating a platform for the recruitment of repair factors [22,24,25]. Moreover, RNF2 mediates monoubiquitination of H2AX, a step required for phosphorylated H2AX formation and subsequent recruitment of MDC1 and ATM activation, and that TIP60 acetylates H2AX to prime it for UBC13-dependent ubiquitination. In particular, 53BP1 and BRCA1 are recruited to this landing platform and enforce the G2/M checkpoint, significantly influencing the choice of the repair pathway [26,47]. 53BP1 in turn assembles effector proteins such as RIF1, Artemis, etc., to limit the extent of DNA end resection and thereby channel repair toward NHEJ [28,48,49,50,51,52,53,54]. Cullin-RING ligases account for ≈10% of the ubiquitylation observed after DNA damage, operating on targets including EXO1, PCNA, and CENPs [31]. In addition, UBR5-mediated ubiquitination of ATMIN at lysine 238 promotes ATM–MRN signaling and checkpoint activation [29]. Overall, DSBs trigger dynamic ubiquitylation of adjacent chromatin areas, and ubiquitin-dependent signaling plays a key role in determining DSB repair pathway choice by modifying NHEJ and HR components and by regulating factors that control DSB end resection [30] (Table 1). Furthermore, the ubiquitin–proteasome pathway is an important cellular mechanism for protein degradation, ensuring the removal of misfolded or damaged proteins, thus preventing their accumulation and potential toxicity [21].

#### 3.3.2. SUMOylation

SUMO signaling plays an important role in the tightly controlled protein choreography at DSB sites, and its deregulation impairs genome stability [20,34,38]. SUMOylation, the process of attaching SUMO chains to other proteins, influences DNA repair processes by promoting the ubiquitin-dependent degradation of SUMOylated DDR factors. This process supports the regulation of the assembly and disassembly of repair factors at DNA damage sites, which is crucial for rapid and efficient DDR [20,34]. In the context of radiation-induced DSBs, SUMO protease SENP7 has been shown to desumoylate KAP1 (KRAB domain-associated protein 1), a key factor in maintaining heterochromatin structure [36]. KAP1 is highly autosumoylated through its intrinsic SUMO E3 ligase activity, providing binding sites for CHD3 (chromodomain helicase DNA-binding protein 3), a component of the NuRD (nucleosome remodeling and deacetylase) chromatin remodeling complex that mediates chromatin compaction. In response to DSBs, KAP1 undergoes ATM-dependent phosphorylation in heterochromatin, triggering the release of CHD3 and chromatin relaxation, thus facilitating access of the repair machinery to breaks in otherwise compacted chromatin regions [55]. SUMO conjugation observed in response to DNA breakage promotes accumulation of ubiquitin chains on damaged chromatin and is required for the efficient recruitment of ubiquitin-dependent repair factors such as MDC1, TP53BP1, BRCA1, RPA, and EXO1 [33,35,39]. In both NHEJ and HR, SUMOylation contributes to the recruitment and coordinated removal of repair factors and modifies key DDR proteins such as p53, PCNA, and Rad52, affecting their localization, stability, and activity [32,33,35,37] (Table 1). Moreover, SUMOylation can serve as signal for subsequent ubiquitination, often leading to altered protein interactions or proteasomal degradation [35].

#### 3.3.3. PARylation

In response to DNA damage, poly(ADP-ribose)(PAR) polymerases (PARP) synthesize ADP-ribose polymers, which then serve as docking sites for proteins with PAR-binding domains. This recruitment of proteins to the site of DNA damage facilitates DNA repair processes [43,56,57,58]. PAR-dependent events are crucial for the cellular response to DNA damage, particularly in the repair of single-strand breaks (SSBs) and DSBs, and in maintaining the stability of disrupted replication forks. These events involve the rapid and transient synthesis of PAR chains at DNA damage sites, forming a platform to recruit various proteins involved in DNA repair and chromatin remodeling [40,41,42,44] (Table 1). The relative contribution of each of these components to reliable DNA repair is not yet well understood and may depend on the type and complexity of the damage as well as on the overall damage burden, together with cell cycle phase and local chromatin environment. Together, signaling by ubiquitylation, SUMOylation and PARylation orchestrates cellular responses to DSBs at multiple levels and with intensive crosstalks to promote precise repair of DNA lesions and protect genome integrity.

### 3.4. Radiation-Induced Changes in DNA Methylation

#### 3.4.1. Global DNA Methylation

First studies on the effects of IR exposure on DNA methylation date back to the late 1980s, when a dose-dependent decrease in 5-methylcytosine was observed in cultured cell lines after 60Co-γ IR [59]. In following years global DNA methylation was investigated not only in various cell lines [60], but also in different organ tissues in animal models following different fractionation schemes and radiation qualities [61,62,63,64,65,66,67]. Although the results of these early studies were not always conclusive, they generally showed that IR exposure leads to changes in global DNA methylation, with the extent of radiation-induced changes being cell- and tissue-dependent and varying widely between experimental models [68]. Animal studies, particularly in rodent models, have shown that radiation-induced alterations in genomic DNA methylation are not ubiquitous between different organ tissues and tissue-specific cell populations. In contrast, these changes occur in dose-dependent, sex-, and tissue-specific manners and can persist following IR exposure. A recent study investigated long-term changes in DNA methylation in hearts of irradiated rats and blood of breast cancer patients undergoing fractionated radiotherapy [69]. Based on their results, the authors conclude that there is a possible link between long-term changes in DNA methylation and pathophysiology of radiation-induced cardiovascular disease [69]. In animal studies, global DNA methylation and gene expression in gonads of newborn mice was investigated after high- and low-dose IR. Ovarian and testicular examinations suggest that different DNA methylation patterns are dose-dependent-induced, but both high- and low-dose IR before sexual maturity impair gametogenesis and fertility [70]. While strongly altered methylation patterns were observed in germline cells of irradiated gonads, no methylation changes were detectable in primary human fibroblasts (with intact cell cycle checkpoints) after ≤4 Gray irradiation. This suggests that global DNA methylation in normal somatic cells remains relatively stable during the early phase of DNA damage response [71]. In a mouse model with fractionated low-dose IR (5x 0.1 Gy), the extent of DNA damage, global DNA methylation, and DNA methylation mechanisms were analyzed in different brain regions and correlated with behavioral changes. Fractionated low-dose IR resulted in increased DNA damage and alterations of the methylome, most pronounced in hippocampal region, and correlated with functional impairments in behavioral testing [72]. In mouse models of whole-body IR, global hypomethylation and promoter hypermethylation of specific genes were investigated in various organ tissues (kidney, liver, spleen, brain, lung) after acute (0.5 Gy X-ray) and chronic low-dose exposure over 10 days (0.05 Gy/day × 10 days). Chronic low-dose IR led to significant loss of global DNA methylation in all analyzed tissues and tissue-specific promoter hypermethylations [73]. Although the exact mechanisms of radiation-induced methylation in different tissues are not yet fully understood, local promoter methylation is an important hallmark of cancer. Since changes in DNA methylation patterns may influence cell proliferation and differentiation, especially in hierarchically organized tissues with high cell turnover, these radiation-induced epigenetic processes may impair cell functions and lead to altered radiosensitivity (Table 2).

#### 3.4.2. Gene-Specific DNA Methylation

Exogenous stressors such as IR can affect methylation profiles globally, but they can also alter promoter regions of specific genes and thus selectively modify their transcriptional activity. Specific hypo- or hypermethylation of genes often affect circumscribed CpG islands and are associated with the activation or silencing of corresponding genes. Accordingly, transcriptional activation of oncogenes induced by DNA hypomethylation or silencing of tumor suppressor genes induced by DNA hypermethylation are driving mechanisms of oncogenic processes observed in virtually all cancers. Accordingly, abberant promoter methylation of the tumor suppressor gene p16^INK4a^ and O(6)-Methylguanine-DNA Methyltransferase was observed in uranium miners with occupational radon exposure [83]. Epigenetic inactivation by these specific promoter hypermethylation are characteristic features of carcinogenesis in lung cancer [75]. Significant correlations between the extent of this gene-specific hypermethylation and cumulative radon doses were observed among uranium miners [74]. Epigenetic studies found that p16^INK4A^ hypermethylation was clearly higher in lung adenocarcinomas from workers exposed to plutonium at the Russian nuclear facility MAYAK compared with non-worker controls [84]. Additional studies on MAYAK workers with lung adenocarcinomas revealed the hypermethylation of transcription factor GATA5, which is required for proper renewal of differentiated epithelium [74]. In vitro studies on cultured cells demonstrate that irradiated cells acquire epigenetic changes in DNA methylation patterns at different genomic regions, dependent on the time after IR exposure and on the genetic background of the cell [76]. Further analysis of gene-specific DNA methylation patterns following IR exposure show that these differentially methylated genes were enriched in gene ontology categories related to cell cycle, DNA repair, and apoptosis pathways, suggesting possible roles in the cellular response to IR [60]. Genome-wide CpG methylation profiling in radioresistant versus radiosensitive cell lines suggests that epigenetic regulation mechanisms are associated with differential radiosensitivity [76]. Moreover, genome-wide DNA methylation profiling technology identified hypomethylation in CpG islands of cancer-related genes in colorectal cancer cells following IR exposure (≤5 Gy), suggesting that global and gene-specific DNA methylation changes contribute to radiation-induced tumorigenesis [78]. Hypermethylation of CpG islands in the p16^INK4A^ promoter and associated transcriptional silencing was demonstrated in mouse models of radiation-induced thymic lymphoma [77]. Overall, both changes in global DNA methylation patterns and hypo- or hypermethylation of certain cancer-related genes were observed following IR exposure, suggesting that radiation-induced epigenetic changes play crucial roles in tumorigenesis (Table 2). However, the significance of radiation-induced changes in DNA methylation patterns for the radiosensitivity of normal tissues has so far been insufficiently investigated.

#### 3.4.3. DNA Methylation of Repetitive Elements

**Figure 1:**
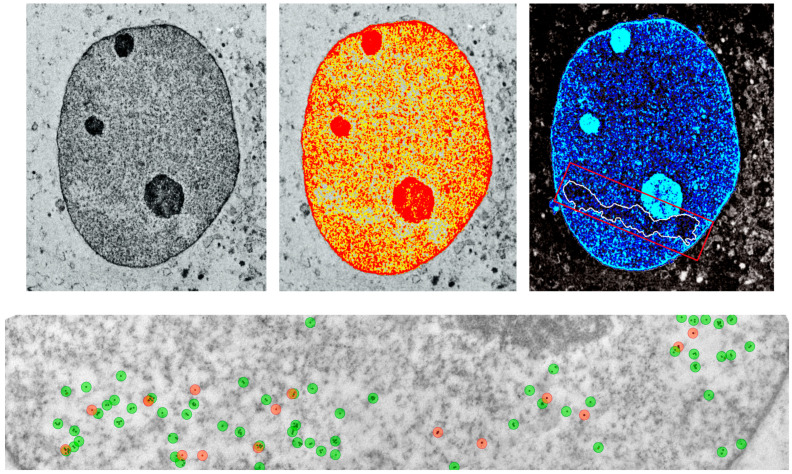
TEM micrographs of a nucleus 5 h after high-LET IR (carbon ions with horizontal beam direction) of human fibroblasts: Due to accumulated DNA damage (10 nm and 6 nm gold beads labeled for pKu70: red, and 53BP1: green, respectively) the chromatin structure along the particle track is massively decondensed.

More than 50% of the mammalian genome is composed of non-coding repetitive elements that have spread throughout the genome during evolution via RNA intermediates in a copy-and-paste mechanism. This family of retrotransposons includes Long Interspersed Nucleotide Element 1 (LINE-1) and Alu elements, whose homotypic clustering forms exclusive nuclear domains that characterize heterochromatin and euchromatin, respectively [80]. Changes in the DNA methylation status of these repetitive elements often lead to their reactivation and retrotransposition, and they have been documented in various cancers and in response to environmental stressors, including IR [68]. In vitro and in vivo studies have demonstrated that alterations in global DNA methylation, at both early and late post-exposure time-points, may stem primarily from these transposable elements [68]. IR of different cell lines and mouse tissues resulted in cell- and tissue-specific, dose- and time-dependent changes in the methylation status of repetitive elements [79,85]. In particular, densely IR with high linear energy transfer (LET) can induce changes in DNA methylation in repetitive elements such as LINE-1 [81]. While low-LET IR has only minor effects on the hypermethylation of LINE-1 elements, high-LET IR with protons and heavy ions in mouse hearts initially leads to hypomethylation, followed by hypermethylation of LINE-1 and other repetitive elements even at late time-points following IR exposure [82,86]. Although numerous studies have identified radiation-induced changes in global and repetitive element-associated DNA methylation, their exact significance for radiosensitivity is still largely unclear. Previous work with transmission electron microscopy (TEM) has shown that high-LET IR causes densely ionizing events along its path, leading to clustered DNA damage and significant disruptions to the chromatin structure [87,88,89,90,91,92,93,94] (compare Figure 1). This disruption, in turn, impairs the ability of cells to repair DNA damage effectively, resulting in more persistent damage and potentially stronger effects on epigenetic modifications. Accordingly, LET-specific epigenetic patterns could have implications for radiation risk assessment in space and cancer therapy with protons and carbon ions, as high-LET radiation more strongly affects epigenetic memory and therefore raises long-term safety concerns. In summary, IR exposure can induce both global and gene-specific DNA methylation changes, with the direction and persistence of these changes depending on dose, exposure pattern, tissue, and biological context. Chronic and fractionated exposures more often result in global hypomethylation, while acute exposures may have minimal or transient effects. Gene-specific hypermethylation, particularly in promoters of DNA repair and cell cycle genes, is a recurring finding and may have functional consequences for disease risk and treatment response (Table 2). The heterogeneity of study designs, models, and reporting standards limits the ability to draw definitive conclusions, highlighting the need for more standardized and mechanistically focused research.

### 3.5. Radiation-Induced Histone Modifications

Detection and repair of radiation-induced DNA damage occurs in the complex chromatin architecture of the cell nucleus. Successful processing of damaged DNA molecules requires chromatin remodeling with the transient reduction in chromatin compaction. During the process of DSB repair, multiple chromatin alterations, especially at the level of post-translational histone modifications, are required to sense damage and facilitate accessibility of the repair machinery. DNA damage sensing and repair is facilitated by hierarchical signaling networks that orchestrate not only dynamic structural changes in chromatin, but recruit DNA repair proteins and regulate their gene expression as well as coordinate cell cycle checkpoints to ensure efficient DNA repair. During these repair processes, histones undergo modifications, including phosphorylation, acetylation, methylation, etc., on their flexible N-terminal tails. Such PTMs form a unique histone code, which represents an epigenetic marking system that regulates chromatin-controlled processes not only in the context of DNA repair, but also has far-reaching consequences regarding cell differentiation and cell fate decision.

#### 3.5.1. Histone Phosphorylation

A well-known radiation-induced histone modification is the phosphorylation of histone variant H2A.X, which is crucially important for DSB repair and for maintenance of genome stability. Phosphorylation of this histone variant at serine 139 (γ-H2A.X) is an early cellular response, occurring in the vicinity of DSB sites within minutes following IR exposure [6]. Phosphorylation of H2A.X at radiation-induced DSBs occurs primarily through ATM or ATR kinases, alters local chromatin structures, and is crucial for the recruitment of signaling/repair proteins to DNA damage sites [95]. Radiation-induced H2A.X phosphorylation leads to the formation of microscopically visible repair foci, which have been extensively used to analyze DNA repair kinetics. H2A.X phosphorylation occurs during all phases of the cell cycle, thus H2A.X phosphorylation is required for both DSB repair pathways, NHEJ and HR [96].

#### 3.5.2. Histone Acetylation

Post-translational acetylation of histones neutralizes positively charged lysine residues, thereby altering intra- and internucleosomal interactions of chromatin fibers to facilitate chromatin decondensation. Acetylation is a dynamic histone marker regulated by the balance between HATs and HDACs [97]. HATs and HDACs transfer an acetyl residue from acetyl-coenzyme A to the ε-amino group of lysine or remove the acetyl group, respectively [98]. Histone acetylation occurs during the initial stage of DNA repair to facilitate chromatin opening and subsequent access of repair proteins to DSB sites [99]. Moreover, histone acetylation direct regulatory pathways sensing and processing DNA damage for repair and may determine the selection of DSB repair pathway choice [100]. By remodeling the chromatin structure, epigenetic modifications cooperate with transcription factors and the translational machinery in fine-tuning gene expression. Our current understanding of histone acetylation in relation to radiosensitivity is still limited, but increased histone acetylation due to treatment with HDAC inhibitors appears to alter radiosensitivity by impairing DNA repair and modifying chromatin structure [101]. In vitro studies on both tumor and normal tissue cell lines observed altered responses to IR with enhanced cell cycle arrest, altered expression of repair proteins, promotion of apoptosis, and decreased cell survival [102,103] (Table 3). In summary, altered histone acetylation during IR exposure appears to be associated with increased radiosensitivity through modulation of important repair and cell cycle processes.

#### 3.5.3. Histone Methylation

Eukaryotic cells must integrate DSB repair signaling and repair via NHEJ and HR pathways with the complexity of local chromatin architectures. Functional chromatin domains differ considerably in patterns of histone modifications and degree of nucleosome packing, thus requiring unique chromatin remodeling complexes to alter local chromatin architectures at individual DSBs. Early events in the DNA damage response may have profound impact on processing of damaged chromatin templates and restructuring nuclear architectures to restore the original epigenetic code and normal cell functionality. Accordingly, histone methylation can significantly influence DNA damage repair, apoptosis, cell cycle, and other biological processes closely related to cellular radiosensitivity [104,106]. During histone methylation, methyl groups are transferred to amino acids of histone proteins at basic residues of arginine, lysine, and histidine. All these amino acids can be monomethylated (me1), arginine and lysine dimethylated (me2), and even lysine trimethylated (me3) through the function of histone methyltransferases (HMTs) and histone demethylases (HDMs) [108]. Common methylation sites associated with DNA damage repair are histone H3 lysine 4 (H3K4), H3K9, H3K27, H3K36, H3K79, and H4K20, with methylation of H3K4, H3K36, and H3K79 being responsible for gene-activating events, while methylation of H3K9, H3K27, and H4K20 is associated with gene-repressing events [107]. Numerous histone modifications are directly involved in the early detection of DNA damage and the opening of chromatin, and at the same time, repressive patterns near DSB damage sites appear to suppress transcription [105]. Several studies suggest that specific histone methylation profiles exist in radioresistant versus radiosensitive tissues, which could represent potential molecular prognostic markers. However, there is considerable evidence that both abnormally high and low histone methylation levels of the same type can promote or reduce cellular radiosensitivity through differential regulation of DNA repair processes [107]. Recent advances in stem cell research suggest that IR alters chromatin structure through local and global changes in histone modifications, which particularly affects the ability of cells either to maintain their stem cell identity or to differentiate into specialized cell types [109]. Overall, histone methylation influences cellular responses to IR mainly by modulating DNA repair and cell cycle checkpoint processes (Table 3). Excellent reviews summarize the regulatory mechanisms and specific effects of histone methylation on cellular radiosensitivity [107].

### 3.6. Radiation-Induced Modulation of Non-Coding RNA Expression

Since their discovery in the early 1990s, non-coding RNA expression has emerged as important modulator in many cellular pathways, including cell proliferation, differentiation, and programmed cell death, but the functional significance of specific miRNAs or lncRNAs in the radiation-induced DNA damage response is only gradually being elucidated. Through interaction with DNA, RNA, and proteins, non-coding RNAs can regulate the structure and function of chromatin, gene transcription, mRNAs (including splicing, turnover and translation), and signaling pathways [8]. In the context of radiation response, non-coding RNAs play an important role in transcriptional and post-transcriptional regulation and in the modification of epigenetic marks. At the transcriptional level, non-coding RNAs can regulate the expression of protein-encoding genes by interfering with transcriptional mechanisms, regulating the enhancers and promoters, interacting with RNA-binding proteins and transcription factors, and regulating the transcriptional programs. At the post-transcriptional level, non-coding RNAs can regulate the precursor processing, transport, and translation of mRNA through direct interactions with regulatory proteins, and can affect cell cycle progression and cell differentiation. At the epigenetic level, non-coding RNAs can regulate various modification processes such as DNA methylation, thereby remodeling chromatin structure and influencing the level of gene expression. A number of studies have examined the general and specific effects of miRNA perturbation in different cell types exposed to IR, as reviewed in [110]. Initial studies have shown that the expression levels of several miRNAs change significantly upon irradiation and suggested specific roles of non-coding RNA expression in DNA repair and cellular radiosensitivity [85,111]. Numerous studies have shown that certain miRNAs alter radiosensitivity by modulating mechanisms associated with apoptosis, cell cycle progression, ROS formation, and epithelial–mesenchymal transition [112] (Table 4). Moreover, there is growing evidence that non-coding microRNAs play critical roles in regulating the IR response by modulating the expression of key genes in cellular processes and can act as radiosensitizers or radioresistance factors, depending on their targets.

Upon IR, miR-34a is rapidly upregulated in cancer and normal cells and modulates different biological processes involved in cellular radiation response by targeting genes such as c-Myc expression [113,124]. Measurement of miR-34a expression level prior to radiotherapy might predict normal tissue toxicity, or tumor radioresistance, suitable for tailoring personalized treatment and improving its efficiency [114]. However, these radiation-induced expression changes do not correlate with the absorbed dose [125]. MiR-21, often overexpressed following IR exposure [115,116], can act as cancer-promoting microRNA that negatively regulates tumor suppressor pathways (PDCD4, PTEN, RECK) that are involved in apoptosis, cell growth, and DNA repair [117,118]. This leads to increased cell survival and radioresistance in cancer cells by affecting the PI3K/AKT pathway [119,120]. By targeting the anti-apoptotic gene BCL2, miR-16 can enhance the effectiveness of radiation therapy by sensitizing cells to radiation-induced apoptosis [121,126]. MiR-155 modulates DDR by targeting RAD51, a key player in the HR pathway [122], thereby either enhancing or suppressing repair depending on the cellular context. Collectively, these miRNAs function as fine-tuners of the DDR by targeting critical regulators of DNA repair, survival pathways as well as radiation-induced senescence [123]. Understanding their roles offers promising opportunities for developing miRNA-based radiosensitizers or radioprotectors in cancer therapy.

### 3.7. Radiation-Induced Incorporation of Histone Variants

Radiation-induced incorporation of histone variants involves the replacement of canonical histones with specialized variants, leading to significant changes in chromatin structure and influencing various biological processes. Among canonical histones, the H2A family has the most extensive repertoire of variants [127]. H2A variants exhibit a broad spectrum of sequence identity compared to canonical H2A, with some differing by only a few amino acids (e.g., H2A.X, H2A.J). These variants possess unique structural and functional properties that contribute specifically to chromatin organization and DNA repair mechanisms [128]. H2A.X differs from canonical H2A primarily by the presence of an SQ motif in the C-terminal region, which is phosphorylated at the S139 residue to form γ-H2A.X during the DDR [6]. This phosphorylation event destabilizes the nucleosome by impairing the binding of linker histone H1 to DNA entry and exit sites, thereby facilitating the recruitment of DNA repair proteins and activating signaling cascades at sites of damage [6,129]. This early DDR event occurs shortly after DSB induction. Loss or deficiency of γ-H2A.X reduces the efficiency of DSB repair, leading to increased radiosensitivity [130]. Besides phosphorylation to γ-H2A.X, other radiation-induced modifications of H2A.X such as ubiquitination and acetylation are dynamically regulated during the DDR, influencing chromatin structure and repair factor recruitment [16]. Furthermore, insufficient H2A.X function impairing genomic stability has been associated with increased tumor susceptibility [131]. Like H2A.X, the histone variant H2A.J contains an SQ motif in the C-terminal domain, which can be phosphorylated upon DNA damage and likely plays a crucial role in the DDR following IR exposure [132]. Histone variant H2A.J was originally discovered in association with replicative and premature senescence [133]. H2A.J was shown to critically modulate the SASP by activating DNA damage-specific inflammatory pathways [134]. At the structural level, incorporation of H2A.J appears to weaken the interaction between H1 and linker DNA, leading to increased chromatin accessibility with increased expression of STAT/IRF transcription factors and transcriptional activation of interferon-stimulated genes during senescence [132]. Electron microscopic studies have shown that H2A.J marks persistent DNA damage and modulates the SASP during radiation-induced senescence [133]. Following IR exposure, H2A.J incorporation affects chromatin organization, accessibility, and transcription factor recruitment, modulating inflammatory gene expression and SASP secretome [135,136]. However, H2A.J overexpression can promote radioresistance and oncogenic transformation by impairing SAHF formation and activating WNT16 signaling [137]. Further studies are needed to understand how H2A.J regulates chromatin structure dynamics and gene expression, and how it is involved in the overall DDR of different tissues following IR exposure.

Other histone variants incorporated following IR exposure include H2A.Z, MacroH2A, and H3.3, which are often incorporated at sites of DNA damage and in active chromatin regions. H2A.Z is rapidly exchanged at DSB sites and may promote chromatin destabilization, making DNA more accessible for repair factors. H2A.Z incorporation was shown to be crucial for efficient HR and NHEJ [138]. Although MacroH2A is less studied in the context of IR, macroH2A is generally involved in chromatin compaction and gene repression [139]. Specifically, macroH2A1.1 is involved in limiting chromatin dynamics and regulating PARP1 activity during DDR, while macroH2A1.2 is implicated in regulating DNA repair pathways and maintaining genome stability [140]. Following IR exposure, H3.3 incorporation is associated with chromatin remodeling, particularly in transcriptionally active regions [141]. Enhancer regions exhibit high histone H3.3 turnover, which changes during cellular differentiation [142]. H3.3 mutations impair DNA repair and promote cGAS/STING-mediated immune responses in pediatric high-grade glioma models [143]. Overall, these histone variants are crucial for the orchestration of chromatin and DDR dynamics following IR exposure (Table 5). In-depth mechanistic and translational research studies are needed to characterize in detail these epigenetic mechanisms involved in cellular radiation responses.

## 4. Radiation-Induced Epigenetic Changes and Premature Senescence

As a biological consequence of IR exposure, DNA damage occurs in various forms, such as SSBs and DSBs, some of which can be clustered into complex lesions. Detection and processing of DNA damage occurs within the complex organization of nuclear chromatin [144]. Coordinated interactions of chromatin-modifying factors with DNA damage signaling and repair networks contribute to downstream mechanisms leading to cell cycle arrest, cell survival, or various forms of cell death. Following IR exposure, cells may re-enter the cell cycle or permanently lose their ability to proliferate. This cellular process of premature senescence is triggered by radiation-induced DNA damage, and can occur independently of telomere dysfunction [145]. Senescent cells are arrested in the G0/G1 phase of the cell cycle and are unable to enter the S-phase after mitogenic stimulation. Senescent cells remain viable and metabolically active over long time periods, but show typical morphological changes, such as flattened, enlarged cells and cell nuclei, as well as vacuole-rich cytoplasm. In recent years it has been shown that fundamental changes in the chromatin structure occur during senescence progression [146,147]. Epigenetic hallmarks of cellular senescence are general loss of canonical histone proteins, incorporation of histone variants, such as H2A.J [134], H3.3, and macroH2A [128,148], altered DNA methylation pattern (global hypomethylation and focal hypermethylation), as well as an imbalance between activating and repressive histone modifications [149]. Characteristic changes in the chromatin organization are the senescence-associated heterochromatin foci (SAHF), reflecting dense chromatin foci (visible in DAPI staining), which are characterized by repressive histone modifications [150]. Accordingly, senescent cells are characterized by global restructuring of the chromatin and altered gene expression profiles [151]. In particular, senescent cells show the senescence-associated secretory phenotype (SASP) with the production of pro-inflammatory factors. In recent publications on radiation-induced senescence, high-resolution electron microscopy techniques revealed that the destabilization of nuclear membranes (as result of the lamin-B1 loss) leads to massive chromatin restructuring with the formation of SAHFs. In addition, chromatin fragments emerge from the cell nucleus into the cytoplasm and activate the cGAS-STING interferon signaling pathway, thereby inducing the SASP [152]. Depending on the cell type, senescent cells show dramatic changes in their secretome over time and strongly influence intercellular communication in their environment of multicellular tissues [153]. The resulting cellular interactions not only regulate the function of differentiated cells but are also important for the growth and development of tissue-specific stem and progenitor cells in their specific microenvironment. IR exposure can trigger intense stress signals, so that not only irradiated but also non-irradiated cells in the neighborhood are affected by the secretion of SASP mediators, leading to a broad spectrum of bystander reactions. In the context of radiation-induced premature senescence, the initial DNA damage leads via complex epigenetic processes to a significantly altered chromatin structure, with functional effects on the regulation of transcription [135]. Cellular senescence is a complex, multi-stage biological process in which cells can undergo different stages from initiation to complete senescence in response to IR exposure. Even in early stages of senescence development, altered chromatin structures may have profound effects on the transcription and function of irradiated cells through multiple epigenetic mechanisms. Increasing evidence suggests that radiation-associated senescent cells disrupt intercellular signaling in the microenvironment by secreting pro-inflammatory and fibrogenic mediators, thereby modulating tissue responses such as inflammation and fibrosis.

## 5. Radiation-Induced Epigenetic Changes and Individual Radiosensitivity

Epigenetic modifications are the primary drivers of the aging process. With increasing age, the body’s resilience decreases, making it more susceptible to age-related diseases such as neurodegenerative diseases and cancer. This aging process is accompanied by a gradual decline in organismal functions and characterized by numerous epigenetic changes [154]. In general, the epigenetic effects of IR exposure in various organ tissues appear to be significantly more pronounced in undifferentiated stem cells and proliferating progenitor cells than in differentiated cell populations [12]. This observation corresponds to the law of Bergonié and Tribondeau, which states that the radiosensitivity of normal tissues is directly proportional to the number of undifferentiated cells in the tissue, their mitotic activity, and their proliferation time [155]. This fundamental law of radiobiology applies particularly to embryos, which are extremely sensitive to IR exposure in the early stages of their development. Since IR induces epigenetic reprogramming, particularly in stem and progenitor cells, it seems logical that not only radiation-induced DNA damage, but also radiation-associated epigenetic processes are responsible for this increased radiosensitivity of embryonic and fetal organisms. Increasing data demonstrate that IR exposure of organ tissues leads to epigenetic reprogramming, particularly in undifferentiated stem and progenitor cells, which can directly and dramatically affect stem cell functions [12]. Tissue-specific stem/progenitor cells play a crucial role in tissue homeostasis of various organs, even in adulthood. They are generally defined as non-specialized cells with the capacity for self-renewal through cell division and the potency to develop into more differentiated cell types. Accordingly, the genome and epigenome of stem/progenitor cells must be particularly well protected to ensure the functional integrity of the stem cell compartment in each organ tissue.

Genotoxic damage caused by IR can disrupt tissue homeostasis in organs, particularly by killing proliferating cells. However, radiation-induced epigenetic consequences can also lead to the loss of tissue homeostasis through the decline of stem cell functions and premature exhaustion of stem cell populations [156]. Overall, these genetic and epigenetic alterations result in the loss of tissue functionality and reduced regenerative potential, clearly more pronounced following IR exposure. The complex molecular and cellular mechanisms underlying these radiation-induced changes in organ tissues are only gradually being elucidated. In addition to the aging process, numerous environmental and lifestyle factors can induce alterations in the epigenome. Studies point to high alcohol and cigarette consumption, poor diet, lack of exercise, and psychological stress as harmful lifestyle factors that cause epigenetic changes with negative effects on cell functions [157]. How these epigenetic patterns of certain lifestyle and environmental changes interact with the complex epigenetic processes of IR and potentially amplify the radiation effects is still largely unclear. The enormous progress in the field of epigenome research regarding the comprehension of gene regulation and function will lead to a paradigm shift in our understanding of radiation effects. Not only radiation-induced DNA damage but also the associated epigenetic effects have a decisive influence on the acute and long-term side effects of IR exposure. Radiation-induced epigenetic modifications modulate cellular key functions through their complex interplay and ultimately determine early and late tissue reactions in response to IR exposure. Taken together, these radiation-induced epigenetic changes may contribute to the pathogenesis of acute and chronic radiation effects on organ tissues and ultimately lead to associated diseases, including cancer. The subtle interplay IR exposure and epigenetic adaptive responses, as well as how the inability to adapt can trigger radiation damage still needs to be investigated in detail. Currently, our knowledge about radiation-induced epigenetic mechanisms related to potential health risks for humans is very limited.

## 6. Susceptibility to Radiation-Induced Epigenetic Changes

The hypothesis that radiation-induced epigenetic effects are more pronounced in undifferentiated stem cells and proliferating progenitor cells is supported by several underlying mechanisms. Undifferentiated stem and progenitor cells typically have a more flexible and dynamic epigenetic state compared to fully differentiated cells [158]. Their chromatin is generally more open and accessible, allowing for rapid changes in DNA methylation and histone modifications in response to environmental stimuli [159]. This plasticity makes them more susceptible to epigenetic alterations. Moreover, proliferating cells undergo frequent DNA replication, during which DNA methylation patterns are actively maintained or re-established, but radiation-induced damage during replication can disrupt these processes, leading to aberrant methylation patterns. Similarly, histone modifications are dynamically reconfigured during DNA synthesis, making proliferating cells more vulnerable to epigenetic perturbations. Stem cells often maintain a less stable epigenetic state to preserve their pluripotency and differentiation potential. This inherent flexibility means that external insults like IR can more readily induce lasting epigenetic changes, which might otherwise be tightly regulated in differentiated cells. Stem and progenitor cells may express higher levels or activity of enzymes involved in adding or removing epigenetic marks. IR can influence the activity or recruitment of these enzymes, leading to more pronounced epigenetic alterations in these cell types. In differentiated cells, by contrast, many genes are already stably silenced through well-established epigenetic marks, making their epigenetic landscape less susceptible to radiation-induced modifications. The individual nuclear architecture in different cell types of tissues explains evolutionarily conserved functional features of genomes, including their plasticity and robustness. In summary, the combination of more dynamic and less stable epigenetic landscapes, higher replication rates, and active epigenetic remodeling machinery in undifferentiated and proliferating cells underpins their increased sensitivity to radiation-induced epigenetic changes.

## 7. Potential Challenges and Future Research Directions

In recent decades, radiation research has focused on understanding how IR exposure induces DNA damage and how cells repair this radiation-induced DNA damage. Importantly, epigenetic changes are not only closely linked to the DDR to facilitate efficient DNA repair within the chromatin context but may have much broader implications for cell functions and cell fate decisions by modulating gene expression. Epigenetic alterations can influence whether cells survive, undergo apoptosis, or differentiate, thereby impacting tissue homeostasis and disease progression. Radiation-induced epigenetic changes contribute to cellular reprogramming, critically influence cell functions, and can determine cell fate or cell identity through changes in gene expression. This body of research underscores that epigenetic modifications are integral to the cellular response to radiation, influencing both DNA repair processes and broader cell fate outcomes, and determines the long-term consequences of IR exposure, such as radiation-induced fibrosis or carcinogenesis.

Epigenetic regulation involves a highly interconnected system where multiple modifications and mechanisms work together to control gene expression and cellular responses. Focusing solely on specific epigenetic alterations does not capture the full complexity of the epigenetic regulatory network and limits our understanding of the full biological impact of IR. Radiation-induced genotoxic stress induces cascades of epigenetic changes across different layers—and these epigenetic alterations collectively influence cellular processes such as DNA repair, apoptosis, proliferation, and differentiation. Understanding these effects requires an integrated view that considers how these various epigenetic marks and mechanisms interact over time and across different cell types. A comprehensive perspective that considers the entire epigenetic landscape and its networked interactions is essential to fully grasp how IR influences cellular function and contributes to health outcomes, including carcinogenesis and other radiation-associated long-term effects. Combining data from these different epigenetic levels requires integrative bioinformatics tools to analyze the interactions between different epigenetic layers and link them to functional outcomes. However, variability across platforms and datasets can complicate integration efforts of multi-omics data. Capturing dynamic epigenetic changes over time and within specific cell populations remains challenging, especially in heterogeneous tissues. Single-cell, multi-omics approaches are promising but still face technical and analytical hurdles. Distinguishing whether epigenetic changes are drivers or consequences of radiation effects is complex, and causality can only be determined in functional studies. The development of longitudinal in vivo studies that track epigenetic alterations over time following IR exposure in relevant models will provide insights into the dynamics and persistence of changes. Ultimately, these epigenetic analyses must be combined with other fields such as genomics, proteomics, and systems biology through interdisciplinary approaches to create comprehensive models of radiation response.

## Figures and Tables

**Table 1:** Ubiquitylation, SUMOylation, and PARylation during acute DDR.

Modification Type	Protein Complex	Key Mediators	Regulatory Function	References
**Ubiquitylation**	**RNF8:** Ring Finger Protein 8	H2A, H2A.X: Lys-63 linked polyubiquitination, Lys-48-linked ubiquitination	DDR signaling, recruitment of DNA repair proteins, chromatin remodeling, checkpoint activation, regulator of DDR	Lecker et al., 2006 [21] Doil et al., 2009 [22] Messick & Greenberg 2009 [23] Panier & Durocher 2009 [24] Mattiroli et al., 2012 [25] Jackson & Durocher 2013 [20] Fradet-Turcotte et al., 2013 [26] Pinder et al., 2013 [27] Callen et al., 2013 [28] Zhang et al., 2014 [29] Thorslund et al., 2015 [30] Fouad et al., 2019 [31]
	**RNF168**: Ring Finger Protein 168	H1.2: Ubiquitination (type not specified)	DDR signaling, recruitment of DNA repair proteins (TP53BP1, BRCA1), formation of DNA damage foci, DNA repair	
	**UBR5** Ubiquitin Protein Ligase E3 Component N-Recognin 5	ATMIN (ATM Interactor): Ubiquitination at lysine 238	ATM-MRN signaling, checkpoint activation, regulator of DDR	
	**RNF2** Ring Finger Protein 2	H2A.X: Monoubiquitination	Phosphorylated H2AX formation (γH2AX), MDC1/ATM recruitment, specific tag for epigenetic transcriptional repression	
	**TIP60** Histone acetyltransferase KAT5	H2A und H4: Acetylation-dependent ubiquitination	Modulation of nucleosome-DNA interactions, histone release, chromatin remodeling, transcriptional activation	
	**Cullin-RING ligase** E3 ubiquitin-protein ligase complex	EXO1, PCNA, CENPs: Polyubiquitination (lysine 6/33)	DDR signaling, recruitment of DNA repair proteins, cell cycle progression, signal transduction and transcription	
**SUMOylation**	**SWI/SNF** SWItch/Sucrose Non-Fermentable	ATP-dependent chromatin remodeling by sliding and ejecting nucleosomes	Chromatin relaxation, phosphorylated H2AX (γH2AX), DSB repair, checkpoint maintenance	Hoege et al., 2002 [32] Morris et al., 2009 [33] Bekker-Jensen & Mailand 2011 [34] Galanty et al., 2012 [35] Garvin et al., 2013 [36] Chung & Zhao 2015 [37] Eifler & Vertegaal 2015 [38] Bologna et al., 2015 [39]
	**Brd4** Bromodomain-containing protein 4	Chromatin reader protein that recognizes/binds acetylated histones	DDR signaling, phosphorylated H2AX (γH2AX), insulating regions from DDR by limiting spreading of H2A.X phosphorylation	
	**NuRD** Nucleosome Remodeling and Deacetylase	Multi-protein complex that combines HDAC with nucleosome remodeling activity, typically containing subunits HDAC1/2, CHD3/4	Relaxation of heterochromatin via AP-1, chromatin remodeling	
	**INO80** INO80 Complex ATPase Subunit	Incorporation and removal of alternate histones, e.g. histone variant H2A.Z	Chromatin remodeling, phosphorylated H2AX (γ-H2AX) interactions	
**PARylation**	**PARP1** Poly[ADP-ribose] polymerase 1	By using NAD+ to synthesize poly ADPribose (PAR) and transferring PAR moieties to proteins, including repair factors, chromatin remodelers	Detection of DNA damage, decompaction of chromatin, interaction with multiple DNA repair factors to regulate DNA repair	Ahel et al., 2008 [40] Haince et al., 2008 [41] Gottschalk et al., 2009 [42] Krietsch et al., 2013 [43] Beck et al., 2014 [44]

**Table 2:** DNA methylation following IR exposure.

DNA Methylation Pattern	Target Proteins	Regulatory Function	Biological Relevance	References
**Global DNA methylation**	Genome-wide DNA, germline and somatic cells, various organ tissues	Acute exposure (single or short-term)	Minimal or transient effects (within hours to days)	Kalinich et al., 1989 [59] Tawa et al., 1998 [61] Kovalchuk et al., 2004 [64] Pogribny et al., 2004 [62] Koturbash et al., 2005 [65] Giotopoulos et al., 2006 [66] Loree et al., 2006 [67] Antwih et al., 2013 [60] Wang et al., 2014 [52] Koturbash et al., 2016 [72] Maierhofer et al., 2017 [71] Miousse et al., 2017 [68] Sallam et al., 2022 [69] Nakata et al., 2021 [70]
		Chronic and fractionated exposure (repeated or prolonged over time)	Mixed or time-dependent methylation effects: early hypomethylation followed by normalization or hypermethylation; differences based on dose-rate or fractionation	
**Gene-specific** **DNA methylation**	Promoter regions of specific genes	Modifies transcriptional activity, leading to gene activation or silencing	Impact on cellular responses to IR and carcinogenesis	Lyon et al., 2007 [74] Kontic et al., 2012 [75] Chaudhry& Omaruddin 2012 [76] Antwih et al., 2013 [60] Song et al., 2014 [77] Bae et al., 2015 [78]
	DNA repair (Rad23b, Ddit3) & cell cycle (p16INK4a, MGMT, GATA5)	DNA damage response	Genomic instability, Cancer risk	
	Endothelial function (PGRMC1, UNC119B, RERE, FNDC3B)	Cardiovascular regulation	Cardiovascular disease	
	Immune response (IL5RA, H2AFY, CTSA, LTC4S, RB1)	Immune signaling	Predictive of radiotherapy response	
	Tumor suppressors/oncogenes (RB1, JAK2, BCAM)	Genomic stability	Cancer progression, radioresistance	
**DNA methylation of** **repetitive elements**	Repetitive elements (LINE-1, Alu elements)	Changes can lead to reactivation and retrotransposition	Genomic instability, cancer	Koturbash et al., 2007 [79] de Koning et al., 2011 [80] Goetz et al., 2011 [81] Prior et al., 2016 [82] Miousse et al., 2017 [68]

**Table 3:** Radiation-induced Histone Modifications.

Modification Type	Target Histones	Regulatory Function	Biological Relevance	References
**Histone** **Phosphorylation**	Histone variant H2A.X (serine 139, γ-H2A.X)	Facilitates DNA damage sensing, recruits repair proteins, alters chromatin structure	Crucial for DSB repair, genome stability, formation of repair foci	Rogakou et al., 1998 [6] Pilch et al., 2003 [95] Sedelnikova et al., 2003 [96]
**Histone** **Acetylation**	Histones (lysine residues on N-terminal tails)	Neutralizes positive charge, chromatin condensation, influences repair pathway choice	Facilitates chromatin opening during DNA repair, impacts radiosensitivity	Bird et al., 2002 [99] Legube & Trouche 2003 [97] Zhang et al., 2009 [103] Purrucker et al., 2010 [102] Groselj et al., 2013 [101]
**Histone** **Methylation**	Histone H3 (K4, K9, K27, K36, K79) Histone H4 (K20)	Modulates chromatin accessibility, gene activation/repression, influences DNA repair & cell cycle	Affects DDR, chromatin structure, radiosensitivity, stem cell differentiation	Kouzarides, 2007 [104] Friedl et al., 2012 [105] Gursoy-Yuzugullu et al., 2017 [106] Zhou & Shao 2021 [107]

**Table 4:** Radiation-Induced Modulation of Non-Coding RNA Expression.

Modification	Target Proteins	Regulatory Function	Biological Relevance	References
**miR-34a**	c-Myc	Rapidly upregulated post-IR; modulates genes involved in DDR, including c-Myc	Predicts normal tissue toxicity and tumor radioresistance	He et al., 2017 [111] Lacombe & Zenhausern 2017 [113] Halimi et al., 2016 [114]
**miR-21**	Tumor suppressor PDCD4, PTEN, RECK	Acts as oncomiR; negatively regulates tumor suppressor pathways	Contributes to increased cell survival, radioresistance, affects apoptosis and DNA repair	Shi et al., 2012 [115] Mahmoudi et al., 2022 [116] Jiang et al., 2017 [117] Liu et al., 2019 [118] Gwak et al., 2012 [119] Ghafouri-Fard et al., 2021 [120]
**miR-16**	BCL2	Targets anti-apoptotic BCL2; enhances radiotherapy effectiveness by sensitizing cells to apoptosis	Improves radiosensitivity by promoting apoptosis	Trevisan et al., 2020 [121]
**miR-155**	RAD51	Modulates DDR; influences HR pathway depending on context	Fine-tunes DNA repair, impacts radiation sensitivity or resistance	Gasparini et al., 2014 [122] Wang et al., 2011 [123]

**Table 5:** Radiation-induced Incorporation of Histone Variants.

Histone Variant	Modification by IR	Regulatory Function	Biological Relevance	References
**H2A.X**	Phosphorylation at S139 (γ-H2A.X); ubiquitination, acetylation	Phosphorylation destabilizes nucleosomes, recruits repair proteins, activates DDR	Early DSB marker; essential for efficient DSB repair; loss increases radiosensitivity and impairs genome stability	Rogakou et al., 1998 [6] Celeste et al., 2002 [130] Ikura et al., 2015 [16] Bassing et al., 2002 [131]
**H2A.J**	Phosphorylation at SQ motif; incorporation increases following IR exposure	Modulates chromatin accessibility, promotes inflammatory gene expression	radiation-induced senescence, inflammation, immune responses; overexpression can promote radioresistance and oncogenic transformation	Mangelinck et al., 2020 [132] Contrepois et al., 2017 [134] Isermann et al., 2020 [133] Freyter et al., 2024 [137]
**H2A.Z**	Rapid exchange at DSB sites	Promotes chromatin destabilization, increases accessibility for repair factors	Facilitates HR and NHEJ; crucial for efficient DNA repair	Xu et al., 2012 [138]
**MacroH2A**	Involved in chromatin compaction; regulation of PARP1	Reduces chromatin accessibility and promotes gene repression	Modulates chromatin dynamics; involved in DNA repair and cell survival	Sun & Bernstein, 2019 [139] Ruiz et al., 2019 [140]
**H3.3**	Incorporation during chromatin remodeling; increased at active regions	Associated with active transcription; facilitates chromatin accessibility	Impacts DNA repair efficiency; mutations impair repair and activate immune responses	Deaton et al., 2016 [142] Haase et al., 2022 [143]

## Abbreviations

ATM	Ataxia Telangiectasia-Mutated
ATR	Ataxia Telangiectasia- and Rad3-related
BCL2	B-Cell CLL/Lymphoma 2
BRCA1	Breast Cancer Type 1 Susceptibility Protein
CENPS	Centromere Protein S
CHD3	Chromodomain Helicase DNA-Binding Protein 3
DDR	DNA damage response
DNA	DeoxyRibonucleic Acid
DNMTs	DNA MethylTransferases
DSB	Double-Strand Break
EXO1	Exonuclease 1
GATA5	GATA-Binding Protein 5
Gy	Gray
HATs	Histone AcetylTransferases
HMTs	Histone MethylTransferases
INO80	INO80 Complex ATPase Subunit
IR	Ionizing Radiation
HR	Homologous Recombination
KAP1	KRAB domain-associated protein 1
LINE-1	Long Interspersed Nucleotide Element 1
LET	linear energy transfer
MDC1	Mediator Of DNA Damage Checkpoint 1
MRN	MRE11-RAD50-NBS1 complex
NHEJ	Non-Homologous End-Joining
PCNA	Proliferating Cell Nuclear Antigen
PTMs	Post-Translational Modifications
PI3K	Phosphatidylinositol-4,5-Bisphosphate 3-Kinase
AKT	AKT Serine/Threonine Kinase
RAD51	DNA Repair Protein RAD51 Homolog
RAD52	DNA Repair Protein RAD52 Homolog
RIF1	Replication Timing Regulatory Factor 1
RNF2/8/168	Ring Finger Protein 2/8/168
ROS	Reactive Oxygen Species
RPA1	Replication Protein A1
SAHF	Senescence-Associated Heterochromatin Foci
SASP	Senescence-Associated Secretory Phenotype
SSB	Single-Strand Break
STAT3	Signal Transducer And Activator Of Transcription 3
Sv	Sievert
IRF1	Interferon Regulatory Factor 1
SENP7	SUMO Specific Peptidase 7
SWI/SNF	Switch/Sucrose Non-Fermentable
TP53BP1	Tumor Protein P53-Binding Protein 1
WNT16	Wnt Family Member 16

## References

[1] Li G., Zhu P. (2015). Structure and organization of chromatin fiber in the nucleus. FEBS Lett..

[2] Cheema M.S., Ausio J. (2015). The Structural Determinants behind the Epigenetic Role of Histone Variants. Genes.

[3] Li G., Reinberg D. (2011). Chromatin higher-order structures and gene regulation. Curr. Opin. Genet. Dev..

[4] Mattei A.L., Bailly N., Meissner A. (2022). DNA methylation: A historical perspective. Trends Genet..

[5] Liu R., Wu J., Guo H., Yao W., Li S., Lu Y., Jia Y., Liang X., Tang J., Zhang H. (2023). Post-translational modifications of histones: Mechanisms, biological functions, and therapeutic targets. MedComm.

[6] Rogakou E.P., Pilch D.R., Orr A.H., Ivanova V.S., Bonner W.M. (1998). DNA double-stranded breaks induce histone H2AX phosphorylation on serine 139. J. Biol. Chem..

[7] Jenuwein T., Allis C.D. (2001). Translating the histone code. Science.

[8] Wei J.W., Huang K., Yang C., Kang C.S. (2017). Non-coding RNAs as regulators in epigenetics (Review). Oncol. Rep..

[9] Oberdoerffer P., Miller K.M. (2023). Histone H2A variants: Diversifying chromatin to ensure genome integrity. Semin. Cell Dev. Biol..

[10] Jackson S.P., Bartek J. (2009). The DNA-damage response in human biology and disease. Nature.

[11] Vergara X., Manjón A.G., de Haas M., Morris B., Schep R., Leemans C., Friskes A., Beijersbergen R.L., Sanders M.A., Medema R.H. (2024). Widespread chromatin context-dependencies of DNA double-strand break repair proteins. Nat. Commun..

[12] Fabbrizi M.R., Warshowsky K.E., Zobel C.L., Hallahan D.E., Sharma G.G. (2018). Molecular and epigenetic regulatory mechanisms of normal stem cell radiosensitivity. Cell Death Discov..

[13] Price B.D., D’Andrea A.D. (2013). Chromatin remodeling at DNA double-strand breaks. Cell.

[14] Harrod A., Lane K.A., Downs J.A. (2020). The role of the SWI/SNF chromatin remodelling complex in the response to DNA double strand breaks. DNA Repair.

[15] Sun Y., Jiang X., Chen S., Fernandes N., Price B.D. (2005). A role for the Tip60 histone acetyltransferase in the acetylation and activation of ATM. Proc. Natl. Acad. Sci. USA.

[16] Ikura M., Furuya K., Matsuda S., Matsuda R., Shima H., Adachi J., Matsuda T., Shiraki T., Ikura T. (2015). Acetylation of Histone H2AX at Lys 5 by the TIP60 Histone Acetyltransferase Complex Is Essential for the Dynamic Binding of NBS1 to Damaged Chromatin. Mol. Cell. Biol..

[17] van Attikum H., Fritsch O., Hohn B., Gasser S.M. (2004). Recruitment of the INO80 complex by H2A phosphorylation links ATP-dependent chromatin remodeling with DNA double-strand break repair. Cell.

[18] van Attikum H., Fritsch O., Gasser S.M. (2007). Distinct roles for SWR1 and INO80 chromatin remodeling complexes at chromosomal double-strand breaks. EMBO J..

[19] Karl L.A., Peritore M., Galanti L., Pfander B. (2021). DNA Double Strand Break Repair and Its Control by Nucleosome Remodeling. Front Genet..

[20] Jackson S.P., Durocher D. (2013). Regulation of DNA damage responses by ubiquitin and SUMO. Mol. Cell.

[21] Lecker S.H., Goldberg A.L., Mitch W.E. (2006). Protein degradation by the ubiquitin-proteasome pathway in normal and disease states. J. Am. Soc. Nephrol..

[22] Doil C., Mailand N., Bekker-Jensen S., Menard P., Larsen D.H., Pepperkok R., Ellenberg J., Panier S., Durocher D., Bartek J. (2009). RNF168 binds and amplifies ubiquitin conjugates on damaged chromosomes to allow accumulation of repair proteins. Cell.

[23] Messick T.E., Greenberg R.A. (2009). The ubiquitin landscape at DNA double-strand breaks. J. Cell Biol..

[24] Panier S., Durocher D. (2009). Regulatory ubiquitylation in response to DNA double-strand breaks. DNA Repair.

[25] Mattiroli F., Vissers J.H., van Dijk W.J., Ikpa P., Citterio E., Vermeulen W., Marteijn J.A., Sixma T.K. (2012). RNF168 ubiquitinates K13-15 on H2A/H2AX to drive DNA damage signaling. Cell.

[26] Fradet-Turcotte A., Canny M.D., Escribano-Díaz C., Orthwein A., Leung C.C.Y., Huang H., Landry M.-C., Kitevski-LeBlanc J., Noordermeer S.M., Sicheri F. (2013). 53BP1 is a reader of the DNA-damage-induced H2A Lys 15 ubiquitin mark. Nature.

[27] Pinder J.B., Attwood K.M., Dellaire G. (2013). Reading, writing, and repair: The role of ubiquitin and the ubiquitin-like proteins in DNA damage signaling and repair. Front. Genet..

[28] Callen E., Di Virgilio M., Kruhlak M.J., Nieto-Soler M., Wong N., Chen H.-T., Faryabi R.B., Polato F., Santos M., Starnes L.M. (2013). 53BP1 mediates productive and mutagenic DNA repair through distinct phosphoprotein interactions. Cell.

[29] Zhang T., Cronshaw J., Kanu N., Snijders A.P., Behrens A. (2014). UBR5-mediated ubiquitination of ATMIN is required for ionizing radiation-induced ATM signaling and function. Proc. Natl. Acad. Sci. USA.

[30] Thorslund T., Ripplinger A., Hoffmann S., Wild T., Uckelmann M., Villumsen B., Narita T., Sixma T.K., Choudhary C., Bekker-Jensen S. (2015). Histone H1 couples initiation and amplification of ubiquitin signalling after DNA damage. Nature.

[31] Fouad S., Wells O.S., Hill M.A., D’aNgiolella V. (2019). Cullin Ring Ubiquitin Ligases (CRLs) in Cancer: Responses to Ionizing Radiation (IR) Treatment. Front. Physiol..

[32] Hoege C., Pfander B., Moldovan G.L., Pyrowolakis G., Jentsch S. (2002). RAD6-dependent DNA repair is linked to modification of PCNA by ubiquitin and SUMO. Nature.

[33] Morris J.R., Boutell C., Keppler M., Densham R., Weekes D., Alamshah A., Butler L., Galanty Y., Pangon L., Kiuchi T. (2009). The SUMO modification pathway is involved in the BRCA1 response to genotoxic stress. Nature.

[34] Bekker-Jensen S., Mailand N. (2011). The ubiquitin- and SUMO-dependent signaling response to DNA double-strand breaks. FEBS Lett..

[35] Galanty Y., Belotserkovskaya R., Coates J., Jackson S.P. (2012). RNF4, a SUMO-targeted ubiquitin E3 ligase, promotes DNA double-strand break repair. Genes Dev..

[36] Garvin A.J., Densham R.M., A Blair-Reid S., Pratt K.M., Stone H.R., Weekes D., Lawrence K.J., Morris J.R. (2013). The deSUMOylase SENP7 promotes chromatin relaxation for homologous recombination DNA repair. EMBO Rep..

[37] Chung I., Zhao X. (2015). DNA break-induced sumoylation is enabled by collaboration between a SUMO ligase and the ssDNA-binding complex RPA. Genes Dev..

[38] Eifler K., Vertegaal A.C.O. (2015). SUMOylation-Mediated Regulation of Cell Cycle Progression and Cancer. Trends Biochem. Sci..

[39] Bologna S., Altmannova V., Valtorta E., Koenig C., Liberali P., Gentili C., Anrather D., Ammerer G., Pelkmans L., Krejci L. (2015). Sumoylation regulates EXO1 stability and processing of DNA damage. Cell Cycle.

[40] Ahel I., Ahel D., Matsusaka T., Clark A.J., Pines J., Boulton S.J., West S.C. (2008). Poly(ADP-ribose)-binding zinc finger motifs in DNA repair/checkpoint proteins. Nature.

[41] Haince J.-F., McDonald D., Rodrigue A., Déry U., Masson J.-Y., Hendzel M.J., Poirier G.G. (2008). PARP1-dependent kinetics of recruitment of MRE11 and NBS1 proteins to multiple DNA damage sites. J. Biol. Chem..

[42] Gottschalk A.J., Timinszky G., Kong S.E., Jin J., Cai Y., Swanson S.K., Washburn M.P., Florens L., Ladurner A.G., Conaway J.W. (2009). Poly(ADP-ribosyl)ation directs recruitment and activation of an ATP-dependent chromatin remodeler. Proc. Natl. Acad. Sci. USA.

[43] Krietsch J., Rouleau M., Pic É., Ethier C., Dawson T.M., Dawson V.L., Masson J.-Y., Poirier G.G., Gagné J.-P. (2013). Reprogramming cellular events by poly(ADP-ribose)-binding proteins. Mol. Asp. Med..

[44] Beck C., Robert I., Reina-San-Martin B., Schreiber V., Dantzer F. (2014). Poly(ADP-ribose) polymerases in double-strand break repair: Focus on PARP1, PARP2 and PARP3. Exp. Cell Res..

[45] Aparicio T., Baer R., Gautier J. (2014). DNA double-strand break repair pathway choice and cancer. DNA Repair.

[46] Chapman J.R., Taylor M.R., Boulton S.J. (2012). Playing the end game: DNA double-strand break repair pathway choice. Mol. Cell.

[47] Panier S., Boulton S.J. (2014). Double-strand break repair: 53BP1 comes into focus. Nat. Rev. Mol. Cell Biol..

[48] Di Virgilio M., Callen E., Yamane A., Zhang W., Jankovic M., Gitlin A.D., Feldhahn N., Resch W., Oliveira T.Y., Chait B.T. (2013). Rif1 prevents resection of DNA breaks and promotes immunoglobulin class switching. Science.

[49] Chapman J.R., Barral P., Vannier J.-B., Borel V., Steger M., Tomas-Loba A., Sartori A.A., Adams I.R., Batista F.D., Boulton S.J. (2013). RIF1 is essential for 53BP1-dependent nonhomologous end joining and suppression of DNA double-strand break resection. Mol. Cell.

[50] Escribano-Díaz C., Orthwein A., Fradet-Turcotte A., Xing M., Young J.T., Tkáč J., Cook M.A., Rosebrock A.P., Munro M., Canny M.D. (2013). A cell cycle-dependent regulatory circuit composed of 53BP1-RIF1 and BRCA1-CtIP controls DNA repair pathway choice. Mol. Cell.

[51] Zimmermann M., Lottersberger F., Buonomo S.B., Sfeir A., de Lange T. (2013). 53BP1 regulates DSB repair using Rif1 to control 5′ end resection. Science.

[52] Wang J., Aroumougame A., Lobrich M., Li Y., Chen D., Chen J., Gong Z. (2014). PTIP associates with Artemis to dictate DNA repair pathway choice. Genes Dev..

[53] Boersma V., Moatti N., Segura-Bayona S., Peuscher M.H., van der Torre J., Wevers B.A., Orthwein A., Durocher D., Jacobs J.J.L. (2015). MAD2L2 controls DNA repair at telomeres and DNA breaks by inhibiting 5′ end resection. Nature.

[54] Xu G., Chapman J.R., Brandsma I., Yuan J., Mistrik M., Bouwman P., Bartkova J., Gogola E., Warmerdam D., Barazas M. (2015). REV7 counteracts DNA double-strand break resection and affects PARP inhibition. Nature.

[55] Ziv Y., Bielopolski D., Galanty Y., Lukas C., Taya Y., Schultz D.C., Lukas J., Bekker-Jensen S., Bartek J., Shiloh Y. (2006). Chromatin relaxation in response to DNA double-strand breaks is modulated by a novel ATM- and KAP-1 dependent pathway. Nat. Cell Biol..

[56] Teloni F., Altmeyer M. (2016). Readers of poly(ADP-ribose): Designed to be fit for purpose. Nucleic Acids Res..

[57] Golia B., Singh H.R., Timinszky G. (2015). Poly-ADP-ribosylation signaling during DNA damage repair. Front. Biosci..

[58] Izhar L., Adamson B., Ciccia A., Lewis J., Pontano-Vaites L., Leng Y., Liang A.C., Westbrook T.F., Harper J.W., Elledge S.J. (2015). A Systematic Analysis of Factors Localized to Damaged Chromatin Reveals PARP-Dependent Recruitment of Transcription Factors. Cell Rep..

[59] Kalinich J.F., Catravas G.N., Snyder S.L. (1989). The effect of gamma radiation on DNA methylation. Radiat Res..

[60] Antwih D.A., Gabbara K.M., Lancaster W.D., Ruden D.M., Zielske S.P. (2013). Radiation-induced epigenetic DNA methylation modification of radiation-response pathways. Epigenetics.

[61] Tawa R., Kimura Y., Komura J.-I., Miyamura Y., Kurishita A., Sasaki M.S., Sakurai H., Ono T. (1998). Effects of X-ray irradiation on genomic DNA methylation levels in mouse tissues. J. Radiat. Res..

[62] Pogribny I., Raiche J., Slovack M., Kovalchuk O. (2004). Dose-dependence, sex- and tissue-specificity, and persistence of radiation-induced genomic DNA methylation changes. Biochem. Biophys. Res. Commun..

[63] Pogribny I., Koturbash I., Tryndyak V., Hudson D., Stevenson S.M., Sedelnikova O., Bonner W., Kovalchuk O. (2005). Fractionated low-dose radiation exposure leads to accumulation of DNA damage and profound alterations in DNA and histone methylation in the murine thymus. Mol. Cancer Res..

[64] Kovalchuk O., Burke P., Besplug J., Slovack M., Filkowski J., Pogribny I. (2004). Methylation changes in muscle and liver tissues of male and female mice exposed to acute and chronic low-dose X-ray-irradiation. Mutat. Res..

[65] Koturbash I., Pogribny I., Kovalchuk O. (2005). Stable loss of global DNA methylation in the radiation-target tissue--a possible mechanism contributing to radiation carcinogenesis?. Biochem. Biophys Res. Commun..

[66] Giotopoulos G., McCormick C., Cole C., Zanker A., Jawad M., Brown R., Plumb M. (2006). DNA methylation during mouse hemopoietic differentiation and radiation-induced leukemia. Exp. Hematol..

[67] Loree J., Koturbash I., Kutanzi K., Baker M., Pogribny I., Kovalchuk O. (2006). Radiation-induced molecular changes in rat mammary tissue: Possible implications for radiation-induced carcinogenesis. Int. J. Radiat. Biol..

[68] Miousse I.R., Kutanzi K.R., Koturbash I. (2017). Effects of ionizing radiation on DNA methylation: From experimental biology to clinical applications. Int. J. Radiat. Biol..

[69] Sallam M., Mysara M., Benotmane M.A., Tamarat R., Santos S.C.R., Crijns A.P.G., Spoor D., Van Nieuwerburgh F., Deforce D., Baatout S. (2022). DNA Methylation Alterations in Fractionally Irradiated Rats and Breast Cancer Patients Receiving Radiotherapy. Int. J. Mol. Sci..

[70] Nakata A., Sato K., Fujishima Y., Ting V.G.S., Nakayama K., Ariyoshi K., Tsuruoka C., Shang Y., Iizuka D., Kakinuma S. (2021). Evaluation of Global DNA Methylation and Gene Expression of Izumo1 and Izumo1r in Gonads after High- and Low-Dose Radiation in Neonatal Mice. Biology.

[71] Maierhofer A., Flunkert J., Dittrich M., Müller T., Schindler D., Nanda I., Haaf T., Amendola R. (2017). Analysis of global DNA methylation changes in primary human fibroblasts in the early phase following X-ray irradiation. PLoS ONE.

[72] Koturbash I., Jadavji N.M., Kutanzi K., Rodriguez-Juarez R., Kogosov D., Metz G.A., Kovalchuk O. (2016). Fractionated low-dose exposure to ionizing radiation leads to DNA damage, epigenetic dysregulation, and behavioral impairment. Environ. Epigenet..

[73] Wang J., Zhang Y., Xu K., Mao X., Xue L., Liu X., Yu H., Chen L., Chu X., Castresana J.S. (2014). Genome-wide screen of DNA methylation changes induced by low dose X-ray radiation in mice. PLoS ONE.

[74] Lyon C.M., Klinge D.M., Liechty K.C., Gentry F.D., March T.H., Kang T., Gilliland F.D., Adamova G., Rusinova G., Telnov V. (2007). Radiation-induced lung adenocarcinoma is associated with increased frequency of genes inactivated by promoter hypermethylation. Radiat. Res..

[75] Kontic M., Stojsic J., Jovanovic D., Bunjevacki V., Ognjanovic S., Kuriger J., Puumala S., Nelson H.H. (2012). Aberrant promoter methylation of CDH13 and MGMT genes is associated with clinicopathologic characteristics of primary non-small-cell lung carcinoma. Clin. Lung Cancer.

[76] Chaudhry M.A., Omaruddin R.A. (2012). Differential DNA methylation alterations in radiation-sensitive and -resistant cells. DNA Cell Biol..

[77] Song W., Liu Y., Liu Y., Zhang C., Yuan B., Zhang L., Sun S., Deng D. (2014). Increased p16 DNA methylation in mouse thymic lymphoma induced by irradiation. PLoS ONE.

[78] Bae J.H., Kim J.G., Heo K., Yang K., Kim T.O., Yi J.M. (2015). Identification of radiation-induced aberrant hypomethylation in colon cancer. BMC Genomics..

[79] Koturbash I., Boyko A., Rodriguez-Juarez R., McDonald R.J., Tryndyak V.P., Kovalchuk I., Pogribny I.P., Kovalchuk O. (2007). Role of epigenetic effectors in maintenance of the long-term persistent bystander effect in spleen in vivo. Carcinogenesis.

[80] de Koning A.P., Gu W., Castoe T.A., Batzer M.A., Pollock D.D. (2011). Repetitive elements may comprise over two-thirds of the human genome. PLoS Genet..

[81] Goetz W., Morgan M.N.M., Baulch J.E. (2011). The effect of radiation quality on genomic DNA methylation profiles in irradiated human cell lines. Radiat. Res..

[82] Prior S., Miousse I.R., Nzabarushimana E., Pathak R., Skinner C., Kutanzi K.R., Allen A.R., Raber J., Tackett A.J., Hauer-Jensen M. (2016). Densely ionizing radiation affects DNA methylation of selective LINE-1 elements. Environ. Res..

[83] Su S., Jin Y., Zhang W., Yang L., Shen Y., Cao Y., Tong J. (2006). Aberrant promoter methylation of p16(INK4a) and O(6)-methylguanine-DNA methyltransferase genes in workers at a Chinese uranium mine. J. Occup. Health.

[84] Belinsky S.A., Klinge D.M., Liechty K.C., March T.H., Kang T., Gilliland F.D., Telnov V. (2004). Plutonium targets the p16 gene for inactivation by promoter hypermethylation in human lung adenocarcinoma. Carcinogenesis.

[85] Aypar U., Morgan W.F., Baulch J.E. (2011). Radiation-induced epigenetic alterations after low and high LET irradiations. Mutat. Res..

[86] Koturbash I., Miousse I.R., Sridharan V., Nzabarushimana E., Skinner C.M., Melnyk S.B., Pavliv O., Hauer-Jensen M., Nelson G.A., Boerma M. (2016). Radiation-induced changes in DNA methylation of repetitive elements in the mouse heart. Mutat. Res..

[87] Rübe C.E., Lorat Y., Schuler N., Schanz S., Wennemuth G., Rübe C. (2011). DNA repair in the context of chromatin: New molecular insights by the nanoscale detection of DNA repair complexes using transmission electron microscopy. DNA Repair.

[88] Timm S., Lorat Y., Jakob B., Taucher-Scholz G., Rübe C.E. (2018). Clustered DNA damage concentrated in particle trajectories causes persistent large-scale rearrangements in chromatin architecture. Radiother. Oncol..

[89] Lorat Y., Brunner C.U., Schanz S., Jakob B., Taucher-Scholz G., Rübe C.E. (2015). Nanoscale analysis of clustered DNA damage after high-LET irradiation by quantitative electron microscopy--the heavy burden to repair. DNA Repair.

[90] Lorat Y., Reindl J., Isermann A., Rübe C., Friedl A.A., Rübe C.E. (2021). Focused Ion Microbeam Irradiation Induces Clustering of DNA Double-Strand Breaks in Heterochromatin Visualized by Nanoscale-Resolution Electron Microscopy. Int. J. Mol. Sci..

[91] Lorat Y., Schanz S., Rube C.E. (2016). Ultrastructural Insights into the Biological Significance of Persisting DNA Damage Foci after Low Doses of Ionizing Radiation. Clin. Cancer Res..

[92] Lorat Y., Schanz S., Schuler N., Wennemuth G., Rube C., Rube C.E. (2012). Beyond repair foci: DNA double-strand break repair in euchromatic and heterochromatic compartments analyzed by transmission electron microscopy. PLoS ONE.

[93] Lorat Y., Timm S., Jakob B., Taucher-Scholz G., Rübe C.E. (2016). Clustered double-strand breaks in heterochromatin perturb DNA repair after high linear energy transfer irradiation. Radiother. Oncol..

[94] Al-Razaq M.A.A., Isermann A., Hecht M., Rübe C.E. (2023). Automated Image Analysis of Transmission Electron Micrographs: Nanoscale Evaluation of Radiation-Induced DNA Damage in the Context of Chromatin. Cells.

[95] Pilch D.R., A Sedelnikova O., Redon C., Celeste A., Nussenzweig A., Bonner W.M. (2003). Characteristics of gamma-H2AX foci at DNA double-strand breaks sites. Biochem. Cell Biol..

[96] Sedelnikova O.A., Pilch D.R., Redon C., Bonner W.M. (2003). Histone H2AX in DNA damage and repair. Cancer Biol. Ther..

[97] Legube G., Trouche D. (2003). Regulating histone acetyltransferases and deacetylases. EMBO Rep..

[98] Shahbazian M.D., Grunstein M. (2007). Functions of site-specific histone acetylation and deacetylation. Annu. Rev. Biochem..

[99] Bird A.W., Yu D.Y., Pray-Grant M.G., Qiu Q., Harmon K.E., Megee P.C., Grant P.A., Smith M.M., Christman M.F. (2002). Acetylation of histone H4 by Esa1 is required for DNA double-strand break repair. Nature.

[100] Ogiwara H., Ui A., Otsuka A., Satoh H., Yokomi I., Nakajima S., Yasui A., Yokota J., Kohno T. (2011). Histone acetylation by CBP and p300 at double-strand break sites facilitates SWI/SNF chromatin remodeling and the recruitment of non-homologous end joining factors. Oncogene.

[101] Groselj B., Sharma N.L., Hamdy F.C., Kerr M., Kiltie A.E. (2013). Histone deacetylase inhibitors as radiosensitisers: Effects on DNA damage signalling and repair. Br. J. Cancer.

[102] Purrucker J.C., Fricke A., Ong M.F., Rube C., Rube C.E., Mahlknecht U. (2010). HDAC inhibition radiosensitizes human normal tissue cells and reduces DNA Double-Strand Break repair capacity. Oncol. Rep..

[103] Zhang F., Zhang T., Teng Z.-H., Zhang R., Wang J.-B., Mei Q.-B. (2009). Sensitization to gamma-irradiation-induced cell cycle arrest and apoptosis by the histone deacetylase inhibitor trichostatin A in non-small cell lung cancer (NSCLC) cells. Cancer Biol. Ther..

[104] Kouzarides T. (2007). Chromatin modifications and their function. Cell.

[105] Friedl A.A., Mazurek B., Seiler D.M. (2012). Radiation-induced alterations in histone modification patterns and their potential impact on short-term radiation effects. Front. Oncol..

[106] Gursoy-Yuzugullu O., Carman C., Serafim R.B., Myronakis M., Valente V., Price B.D. (2017). Epigenetic therapy with inhibitors of histone methylation suppresses DNA damage signaling and increases glioma cell radiosensitivity. Oncotarget.

[107] Zhou Y., Shao C. (2021). Histone methylation can either promote or reduce cellular radiosensitivity by regulating DNA repair pathways. Mutat Res Rev Mutat Res..

[108] Greer E.L., Shi Y. (2012). Histone methylation: A dynamic mark in health, disease and inheritance. Nat. Rev. Genet..

[109] Völker-Albert M., Bronkhorst A., Holdenrieder S., Imhof A. (2020). Histone Modifications in Stem Cell Development and Their Clinical Implications. Stem Cell Rep..

[110] Metheetrairut C., Slack F.J. (2013). MicroRNAs in the ionizing radiation response and in radiotherapy. Curr. Opin. Genet Dev..

[111] Wagner-Ecker M., Schwager C., Wirkner U., Abdollahi A., Huber P.E. (2010). MicroRNA expression after ionizing radiation in human endothelial cells. Radiat Oncol..

[112] Pedroza-Torres A., Romero-Córdoba S.L., Montaño S., Peralta-Zaragoza O., Vélez-Uriza D.E., Arriaga-Canon C., Guajardo-Barreto X., Bautista-Sánchez D., Sosa-León R., Hernández-González O. (2024). Radio-miRs: A comprehensive view of radioresistance-related microRNAs. Genetics.

[113] Lacombe J., Zenhausern F. (2017). Emergence of miR-34a in radiation therapy. Crit. Rev. Oncol. Hematol..

[114] Halimi M., Shahabi A., Moslemi D., Parsian H., Asghari S.M., Sariri R., Yeganeh F., Zabihi E. (2016). Human serum miR-34a as an indicator of exposure to ionizing radiation. Radiat. Environ. Biophys..

[115] Shi Y., Zhang X., Tang X., Wang P., Wang H., Wang Y. (2012). MiR-21 is continually elevated long-term in the brain after exposure to ionizing radiation. Radiat. Res..

[116] Mahmoudi R., Saidijam M., Nikzad S., Tapak L., Alvandi M., Afshar S. (2022). Human exposure to low dose ionizing radiation affects miR-21 and miR-625 expression levels. Mol. Biol. Rep..

[117] Jiang L.-P., He C.-Y., Zhu Z.-T. (2017). Role of microRNA-21 in radiosensitivity in non-small cell lung cancer cells by targeting PDCD4 gene. Oncotarget.

[118] Liu Z., Liang X., Li X., Liu X., Zhu M., Gu Y., Zhou P. (2019). MiRNA-21 functions in ionizing radiation-induced epithelium-to-mesenchymal transition (EMT) by downregulating PTEN. Toxicol. Res..

[119] Gwak H.-S., Kim T.H., Jo G.H., Kim Y.-J., Kwak H.-J., Kim J.H., Yin J., Yoo H., Lee S.H., Park J.B. (2012). Silencing of microRNA-21 confers radio-sensitivity through inhibition of the PI3K/AKT pathway and enhancing autophagy in malignant glioma cell lines. PLoS ONE.

[120] Ghafouri-Fard S., Abak A., Shoorei H., Mohaqiq M., Majidpoor J., Sayad A., Taheri M. (2021). Regulatory role of microRNAs on PTEN signaling. Biomed. Pharmacother..

[121] Trevisan F.A., Rodrigues A.R., Neto F.S.L., Peria F.M., Cirino M.L.d.A., Tirapelli D.P.d.C., Júnior C.G.C. (2020). Apoptosis related microRNAs and MGMT in glioblastoma cell lines submitted to treatments with ionizing radiation and temozolomide. Rep. Pract. Oncol. Radiother..

[122] Gasparini P., Lovat F., Fassan M., Casadei L., Cascione L., Jacob N.K., Carasi S., Palmieri D., Costinean S., Shapiro C.L. (2014). Protective role of miR-155 in breast cancer through RAD51 targeting impairs homologous recombination after irradiation. Proc. Natl. Acad. Sci. USA.

[123] Wang Y., Scheiber M.N., Neumann C., Calin G.A., Zhou D. (2011). MicroRNA regulation of ionizing radiation-induced premature senescence. Int. J. Radiat. Oncol. Biol. Phys..

[124] He X., Yang A., McDonald D.G., Riemer E.C., Vanek K.N., Schulte B.A., Wang G.Y. (2017). MiR-34a modulates ionizing radiation-induced senescence in lung cancer cells. Oncotarget.

[125] Liu C., Zhou C., Gao F., Cai S., Zhang C., Zhao L., Zhao F., Cao F., Lin J., Yang Y. (2011). MiR-34a in age and tissue related radio-sensitivity and serum miR-34a as a novel indicator of radiation injury. Int. J. Biol. Sci..

[126] Cimmino A., Calin G.A., Fabbri M., Iorio M.V., Ferracin M., Shimizu M., Wojcik S.E., Aqeilan R.I., Zupo S., Dono M. (2005). miR-15 and miR-16 induce apoptosis by targeting BCL2. Proc. Natl. Acad. Sci. USA.

[127] Bönisch C., Hake S.B. (2012). Histone H2A variants in nucleosomes and chromatin: More or less stable?. Nucleic Acids Res..

[128] Talbert P.B., Henikoff S. (2021). Histone variants at a glance. J. Cell Sci..

[129] Paull T.T., Rogakou E.P., Yamazaki V., Kirchgessner C.U., Gellert M., Bonner W.M. (2000). A critical role for histone H2AX in recruitment of repair factors to nuclear foci after DNA damage. Curr. Biol..

[130] Celeste A., Petersen S., Romanienko P.J., Fernandez-Capetillo O., Chen H.T., Sedelnikova O.A., Reina-San-Martin B., Coppola V., Meffre E., Difilippantonio M.J. (2002). Genomic instability in mice lacking histone H2AX. Science.

[131] Bassing C.H., Chua K.F., Sekiguchi J., Suh H., Whitlow S.R., Fleming J.C., Monroe B.C., Ciccone D.N., Yan C., Vlasakova K. (2002). Increased ionizing radiation sensitivity and genomic instability in the absence of histone H2AX. Proc. Natl. Acad. Sci. USA.

[132] Mangelinck A., Coudereau C., Courbeyrette R., Ourarhni K., Hamiche A., Redon C., Mann C. (2020). The H2A.J histone variant contributes to Interferon-Stimulated Gene expression in senescence by its weak interaction with H1 and the derepression of repeated DNA sequences. bioRxiv.

[133] Isermann A., Mann C., Rube C.E. (2020). Histone Variant H2A.J Marks Persistent DNA Damage and Triggers the Secretory Phenotype in Radiation-Induced Senescence. Int. J. Mol. Sci..

[134] Contrepois K., Coudereau C., Benayoun B.A., Schuler N., Roux P.-F., Bischof O., Courbeyrette R., Carvalho C., Thuret J.-Y., Ma Z. (2017). Histone variant H2A.J accumulates in senescent cells and promotes inflammatory gene expression. Nat. Commun..

[135] Al-Razaq M.A.A., Freyter B.M., Isermann A., Tewary G., Mangelinck A., Mann C., Rübe C.E. (2023). Role of Histone Variant H2A.J in Fine-Tuning Chromatin Organization for the Establishment of Ionizing Radiation-Induced Senescence. Cells.

[136] Tewary G., Freyter B., Al-Razaq M.A., Auerbach H., Laschke M.W., Kübelbeck T., Kolb A., Mangelinck A., Mann C., Kramer D. (2023). Immunomodulatory Effects of Histone Variant H2A.J in Ionizing Radiation Dermatitis. Int. J. Radiat. Oncol. Biol. Phys..

[137] Freyter B.M., Al-Razaq M.A.A., Hecht M., Rübe C., Rübe C.E. (2024). Studies on Human Cultured Fibroblasts and Cutaneous Squamous Cell Carcinomas Suggest That Overexpression of Histone Variant H2A.J Promotes Radioresistance and Oncogenic Transformation. Genes.

[138] Xu Y., Ayrapetov M.K., Xu C., Gursoy-Yuzugullu O., Hu Y., Price B.D. (2012). Histone H2A.Z controls a critical chromatin remodeling step required for DNA double-strand break repair. Mol. Cell.

[139] Sun Z., Bernstein E. (2019). Histone variant macroH2A: From chromatin deposition to molecular function. Essays Biochem..

[140] Ruiz P.D., Hamilton G.A., Park J.W., Gamble M.J. (2019). MacroH2A1 Regulation of Poly(ADP-Ribose) Synthesis and Stability Prevents Necrosis and Promotes DNA Repair. Mol. Cell. Biol..

[141] Sharma A.K., Bhattacharya S., Khan S.A., Khade B., Gupta S. (2015). Dynamic alteration in H3 serine 10 phosphorylation is G1-phase specific during ionization radiation induced DNA damage response in human cells. Mutat. Res..

[142] Deaton A.M., GómEz-RodrígUez M., Mieczkowski J., Tolstorukov M.Y., Kundu S., I Sadreyev R., Jansen L.E., E Kingston R. (2016). Enhancer regions show high histone H3.3 turnover that changes during differentiation. Elife.

[143] Haase S., Banerjee K., Mujeeb A.A., Hartlage C.S., Núñez F.M., Núñez F.J., Alghamri M.S., Kadiyala P., Carney S., Barissi M.N. (2022). H3.3-G34 mutations impair DNA repair and promote cGAS/STING-mediated immune responses in pediatric high-grade glioma models. J. Clin. Investig..

[144] Seeber A., Gasser S.M. (2017). Chromatin organization and dynamics in double-strand break repair. Curr. Opin Genet Dev..

[145] Kuilman T., Michaloglou C., Mooi W.J., Peeper D.S. (2010). The essence of senescence. Genes Dev..

[146] Chandra T., Ewels P.A., Schoenfelder S., Furlan-Magaril M., Wingett S.W., Kirschner K., Thuret J.-Y., Andrews S., Fraser P., Reik W. (2015). Global reorganization of the nuclear landscape in senescent cells. Cell Rep..

[147] Criscione S.W., Teo Y.V., Neretti N. (2016). The Chromatin Landscape of Cellular Senescence. Trends Genet..

[148] Duarte L.F., Young A.R.J., Wang Z., Wu H.-A., Panda T., Kou Y., Kapoor A., Hasson D., Mills N.R., Ma’aYan A. (2014). Histone H3.3 and its proteolytically processed form drive a cellular senescence programme. Nat. Commun..

[149] Sen P., Shah P.P., Nativio R., Berger S.L. (2016). Epigenetic Mechanisms of Longevity and Aging. Cell.

[150] Corpet A., Stucki M. (2014). Chromatin maintenance and dynamics in senescence: A spotlight on SAHF formation and the epigenome of senescent cells. Chromosoma.

[151] Salama R., Sadaie M., Hoare M., Narita M. (2014). Cellular senescence and its effector programs. Genes Dev..

[152] Freyter B.M., Al-Razaq M.A.A., Isermann A., Dietz A., Azimzadeh O., Hekking L., Gomolka M., Rübe C.E. (2022). Nuclear Fragility in Radiation-Induced Senescence: Blebs and Tubes Visualized by 3D Electron Microscopy. Cells.

[153] Dasgupta N., Arnold R., Equey A., Gandhi A., Adams P.D. (2024). The role of the dynamic epigenetic landscape in senescence: Orchestrating SASP expression. npj Aging.

[154] Horvath S. (2013). DNA methylation age of human tissues and cell types. Genome Biol..

[155] Vogin G., Foray N. (2013). The law of Bergonie and Tribondeau: A nice formula for a first approximation. Int. J. Radiat Biol..

[156] Ermolaeva M., Neri F., Ori A., Rudolph K.L. (2018). Cellular and epigenetic drivers of stem cell ageing. Nat. Rev. Mol. Cell Biol..

[157] Alegria-Torres J.A., Baccarelli A., Bollati V. (2011). Epigenetics and lifestyle. Epigenomics.

[158] Paksa A., Rajagopal J. (2017). The epigenetic basis of cellular plasticity. Curr. Opin. Cell Biol..

[159] Schlesinger S., Meshorer E. (2019). Open Chromatin, Epigenetic Plasticity, and Nuclear Organization in Pluripotency. Dev. Cell.

